# Identification of novel proteins and mRNAs differentially bound to the *Leishmania* Poly(A) Binding Proteins reveals a direct association between PABP1, the RNA-binding protein RBP23 and mRNAs encoding ribosomal proteins

**DOI:** 10.1371/journal.pntd.0009899

**Published:** 2021-10-27

**Authors:** Ludmila A. Assis, Moezio V. C. Santos Filho, Joao R. da Cruz Silva, Maria J. R. Bezerra, Irassandra R. P. U. C. de Aquino, Kleison C. Merlo, Fabiola B. Holetz, Christian M. Probst, Antonio M. Rezende, Barbara Papadopoulou, Tamara D. C. da Costa Lima, Osvaldo P. de Melo Neto

**Affiliations:** 1 Department of Microbiology, Aggeu Magalhães Institute, Oswaldo Cruz Foundation, Recife, Pernambuco, Brazil; 2 Department of Genetics, Federal University of Pernambuco, Recife, Pernambuco, Brazil; 3 Laboratory of Gene Expression Regulation, Carlos Chagas Institute, Oswaldo Cruz Foundation, Curitiba, Paraná, Brazil; 4 CHU de Quebec Research Center and Department of Microbiology-Infectious Disease and Immunology, Laval University, Quebec, Quebec, Canada; 5 University Center Tabosa de Almeida, Caruaru, Pernambuco, Brazil; Bernhard Nocht Institute for Tropical Medicine, Hamburg, Germany, GERMANY

## Abstract

Poly(A) Binding Proteins (PABPs) are major eukaryotic RNA-binding proteins (RBPs) with multiple roles associated with mRNA stability and translation and characterized mainly from multicellular organisms and yeasts. A variable number of PABP homologues are seen in different organisms however the biological reasons for multiple PABPs are generally not well understood. In the unicellular *Leishmania*, dependent on post-transcriptional mechanisms for the control of its gene expression, three distinct PABPs are found, with yet undefined functional distinctions. Here, using RNA-immunoprecipitation sequencing analysis we show that the *Leishmania* PABP1 preferentially associates with mRNAs encoding ribosomal proteins, while PABP2 and PABP3 bind to an overlapping set of mRNAs distinct to those enriched in PABP1. Immunoprecipitation studies combined to mass-spectrometry analysis identified RBPs differentially associated with PABP1 or PABP2, including RBP23 and DRBD2, respectively, that were investigated further. Both RBP23 and DRBD2 bind directly to the three PABPs *in vitro*, but reciprocal experiments confirmed preferential co-immunoprecipitation of PABP1, as well as the EIF4E4/EIF4G3 based translation initiation complex, with RBP23. Other RBP23 binding partners also imply a direct role in translation. DRBD2, in contrast, co-immunoprecipitated with PABP2, PABP3 and with RBPs unrelated to translation. Over 90% of the RBP23-bound mRNAs code for ribosomal proteins, mainly absent from the transcripts co-precipitated with DRBD2. These experiments suggest a novel and specific route for translation of the ribosomal protein mRNAs, mediated by RBP23, PABP1 and the associated EIF4E4/EIF4G3 complex. They also highlight the unique roles that different PABP homologues may have in eukaryotic cells associated with mRNA translation.

## Author summary

Flagellated protozoa belonging to the *Leishmania* genus are the causative agents of the Leishmaniasis, neglected tropical diseases with a worldwide distribution. These parasites are well known for unique molecular mechanisms which include a lack of regulation of mRNA synthesis, generally the major stage for the control of gene expression in eukaryotic cells. Regulation of mRNA translation is therefore critical for survival and adaptation during a life cycle involving multiple life forms. Such regulation is likely dependent on RNA binding proteins (RBPs), such as the Poly-A Binding Protein (PABP) that binds to the 3’ ends of the mRNAs. Three PABPs are found in *Leishmania*, more than in other unicellular organisms, with presumably distinct but undefined roles. Here we identify different mRNAs targets and specific RBPs with whom they interact. We define a specific association between one of the *Leishmania* PABPs, a single RBP and mRNAs encoding ribosomal proteins, an important subset of cellular mRNAs. Our results shed new light on the interactions required for the translation of these mRNAs, highlighting the relevance of regulating their translation in different organisms and the convergence of regulatory mechanisms acting through their 5’ and 3’ ends. The data also expands on the known PABP roles.

## Introduction

The trypanosomatid protozoa constitute a group of parasitic microorganisms which include several species pathogenic to humans, all belonging to the *Leishmania* and *Trypanosoma* genera [1]. These are early divergent eukaryotes characterized by a lack of well-defined RNA polymerase II promoters and constitutive polycistronic transcription. Expression of most of the trypanosomatid genes is regulated post-transcriptionally and it is assumed that this regulation mainly targets mechanisms associated with the processing, transport, stability and translation of mature mRNAs [2–5]. Thus, trypanosomatids emerge as relevant models for the understanding of eukaryotic mechanisms mediating post-transcriptional regulation.

In eukaryotes, a large number of RNA binding proteins (RBPs) recognize and bind specifically to sequences in target mRNAs and are required for their processing, stability, subcellular transport, storage, and translation, with many of these RBPs being specifically associated with regulatory motifs within the untranslated regions (UTRs) of the mRNAs [6–13]. Trypanosomatids have an unusually large number of RBPs belonging to distinct functional families and with different mRNA binding domains. These include those with the RRM (RNA Recognition Motif), ZF (Zinc Finger), PUF (Pumilio) and ALBA (Acetylation Lowers Binding Affinity) domains [14–18]. Many reports have identified differentially regulated RBPs acting as post-transcriptional regulators of gene expression in trypanosomatids, usually binding to sequence elements located within the 3’ UTRs of mature transcripts [5,17–21]. Different mRNAs containing the same motifs and coding for functionally related proteins appear to be similarly regulated [22,23], but detailed mechanisms are generally not well defined.

The cytoplasmic poly(A)-binding proteins, PABPCs or simply PABPs (distinct from the NPABPs, nuclear and more functionally restricted), are the best known and the most abundant of the eukaryotic RBPs. They participate in most, if not all, mRNA associated events, a consequence of their high affinity to the 3’ end adenosine tract, the poly(A) tail, found in almost all eukaryotic mRNAs [24–27]. The PABPs interact with many other proteins, thus participating in various cellular events, with one of the best defined being translation. Within the cytoplasm, PABPs bound to the poly (A) tail interact with the translation initiation complex eIF4F bound to the cap structure of mature mRNAs, thus contributing to the formation of a closed loop structure that might enhance translation or promote translation re-initiation. eIF4F is a heterotrimeric complex of three subunits (eIF4E, eIF4G and the RNA helicase eIF4A) that facilitates the recruitment of the 40S ribosomal subunit to the mRNA. Its eIF4E subunit allows binding to the capped 5’ end of the mRNAs, while a direct interaction between eIF4G and PABP is likely to mediate many of the PABP roles in translation [25,28]. A variable number of PABP homologues can be found in different organisms, some of which may be simultaneously expressed [25,27,29], however the mechanisms by which different homologues can be specifically recruited to distinct sets of mRNAs, and their functional implications, still needs to be better defined.

Three PABP homologues (PABP1, PABP2 and PABP3) were identified in *Leishmania* and other trypanosomatid species with PABP3 found to be lost from the *Trypanosoma* lineage. *Leishmania* PABP2 and PABP3 co-precipitate together and both can migrate to the nucleus after transcription inhibition, while PABP1 remains predominantly in the cytoplasm [30,31]. Similarly, the *Trypanosoma brucei* PABP2, but not PABP1, can also accumulate in the nucleus upon stress conditions [31], reinforcing a nuclear role for PABP2, and PABP2/PABP3, in *Trypanosoma* and *Leishmania* species, respectively. Recent proteomic analysis showed that PABP2 interacts with a wider range of protein partners, while PABP1 co-precipitates with a smaller and distinct set of polypeptides [32,33]. In trypanosomatids, multiple eIF4F complexes have been identified based on several distinct homologues of their eIF4E (six) and eIF4G (five) subunits. The EIF4G3/EIF4E4 based complex, which requires EIF4AI, is so far the only one whose role in translation is better understood. This complex specifically co-immunoprecipitates with PABP1, with a novel and direct interaction between PABP1 and EIF4E4 having been identified [3,34–36]. The PABP1/EIF4E4 interaction is likely to be associated with a common mechanism of regulation, since both proteins were seen to be simultaneously phosphorylated in multiple serine/threonine residues, an event which in *T*. *brucei* was found to be mediated by at least one cell-cycle regulated protein kinase [33,36–38].

The evidence presented so far implies that the *Leishmania*/*Trypanosoma* PABP1 binds to mRNA populations that are distinct than those bound by PABP2 (or PABP2/PABP3 in *Leishmania*), even though both PABPs are abundant proteins that are simultaneously expressed [30–32]. Although there is still no clear explanation for this differential mRNA selection, it is likely to be mediated by RBPs interacting with specific protein partners and mRNA targets. Here, we carried out a search for RBPs specifically associated with each of the *Leishmania infantum* PABP homologues and we selected RBP23 and DRBD2, which co-precipitated with PABP1 or PABP2/PABP3, respectively, for further characterization. Reciprocal co-immunoprecipitation and mass-spectrometry analysis confirmed a strong association between RBP23 and PABP1, as well as with the EIF4E4/EIF4G3 complex, while DRBD2 was found to be more associated with PABP2 and a larger number of different protein partners. RNA immunoprecipitation of individual proteins followed by large-scale RNA-sequencing revealed specific mRNA populations co-precipitating with each PABP homologue as well as RBP23 and DRBD2. Noteworthy is the largely specific co-precipitation of mRNAs encoding ribosomal proteins with both PABP1 and RBP23, with these mRNAs mostly absent from those found with DRBD2 or the two other PABPs. The data presented in this study suggest a new mechanism for the selection of mRNAs encoding ribosomal proteins during translation initiation in trypanosomatids, with those mRNAs being targeted by both PABP1, as part of a larger repertoire of mRNAs, and more specifically by RBP23.

## Results

### Native *Leishmania major* PABPs co-immunoprecipitated distinct sets of mRNAs

Immunoprecipitation (IP) assays have previously shown that *Leishmania* PABP1 does not co-precipitate with either PABP2 or PABP3. In contrast, reciprocal assays have confirmed that PABP2 and PABP3 co-precipitate together [30]. Since multiple PABP molecules are expected to bind to a single mRNA molecule, lack of co-precipitation of PABP1 with the PABP2/PABP3 pair might reflect binding to different mRNA targets. In *T*. *brucei*, lacking PABP3, the PABP1 and PABP2 orthologues differ in their pattern of migration to starvation-stress granules, known to be associated with the bulk of cellular mRNAs. PABP1 and known protein partners are mainly absent from these granules which nevertheless are enriched with PABP2 and associated proteins and this has been interpreted as indicating that while PABP2 binds to most parasite transcripts, PABP1 is most likely associated with a subpopulation of mRNAs [32]. Here, to better define the functional distinctions between the *Leishmania* PABPs, we first investigated their mRNAs targets in *Leishmania major* using purified polyclonal sera generated against the three native paralogues. Following IP assays, co-immunoprecipitated mRNAs were extracted and sequenced by SOLiD. Transcripts with the highest number of reads and log_2_>2 enriched, when compared with the negative control (beads plus extracts but with the sera omitted) (S1 Table), were grouped according to a list of GO functional terms. These were modified as deemed fit to illustrate relevant differences between the proteins. mRNA targets co-precipitated with the three PABPs were generally restricted to transcripts encoding soluble proteins either known to be abundant or encoded by multiple genes. A total of 141 mRNAs were found to be enriched with PABP1, while PABP2 and PABP3 co-precipitated with 142 and 125 enriched transcripts, respectively. Although several putative targets were shared between the three *L*. *major* PABPs, the analysis revealed relevant differences in the percentages of mRNAs assigned to different GO categories (S1 Fig). A greater percentage of mRNAs encoding ribosomal proteins, grouped within the term “structural constituent—ribosome”, were found associated with PABP1 (~60% vs. ~32% and ~31% for PABP2 and PABP3, respectively). In contrast, both PABP2 and PABP3 have a higher proportion of mRNAs encoding proteins with either binding function (~28% and ~30% of the mRNAs for PABP2 and PABP3 vs. ~18% for PABP1) or catalytic (~17% and ~13% of the mRNAs vs. ~6% for PABP1) and transporter (~6% and 8% of the mRNAs vs. ~4% for PABP1) activities. The differences in bound mRNAs between PABP1 and PABP2/PABP3 is even more noticeable when only the top-most, log_2_>4 enriched, messages are considered (S1 Fig). Although 60% of the top-most enriched mRNAs with PABP1 encode ribosomal proteins, these transcripts represent only 22% and 10% of those co-precipitated with PABP2 and PABP3. As a comparison, histone mRNAs represent 15% of the top-most mRNAs with PABP1 while these same messages comprise 39% and 52% of the most enriched transcripts found with PABP2 and PABP3. These results are in agreement with PABP1 binding to distinct mRNAs from those associated with PABP2 or PABP3 and having a marked preference for mRNAs encoding ribosomal proteins, with the latter two proteins having much more similar mRNA association patterns.

### mRNAs co-immunoprecipitated with *L*. *infantum* HA-tagged PABPs

The polyclonal nature of the antibodies used for the set of IPs carried out for the native proteins might be associated with some degree of cross-reactivity and this might be one of the reasons for the overlap seen in mRNAs bound by PABP1 and the PABP2/PABP3 pair. We thus opted to carry out a more refined and independent experiment for the analysis of PABP-bound mRNAs using a previously described *L*. *infantum* cell line expressing PABP1 [33] and newly generated cell lines expressing either PABP2 or PABP3, all having an identical C-terminal HA epitope tag. When compared with the ectopic PABP1-HA, represented by isoforms indicative of phosphorylation events, both HA-tagged PABP2 and PABP3 were visualized as single bands in whole cellular extracts derived from the transfected cell lines (S2 Fig). Cytoplasmic extracts of these cell lines were then prepared after lysis through cavitation, in the absence of any detergent, and then used in IPs performed with monoclonal antibodies immobilized on magnetic beads and directed to the HA tag, therefore avoiding cross-reacting events. As negative controls, parallel IPs were performed using cytoplasmic extracts made from cells having no HA-tagged protein. Co-purified mRNAs were extracted from the IPs and used for Illumina sequencing. The new approach uses different lysis method, antibody and immunoprecipitation/sequencing strategies.

**Fig 1:**
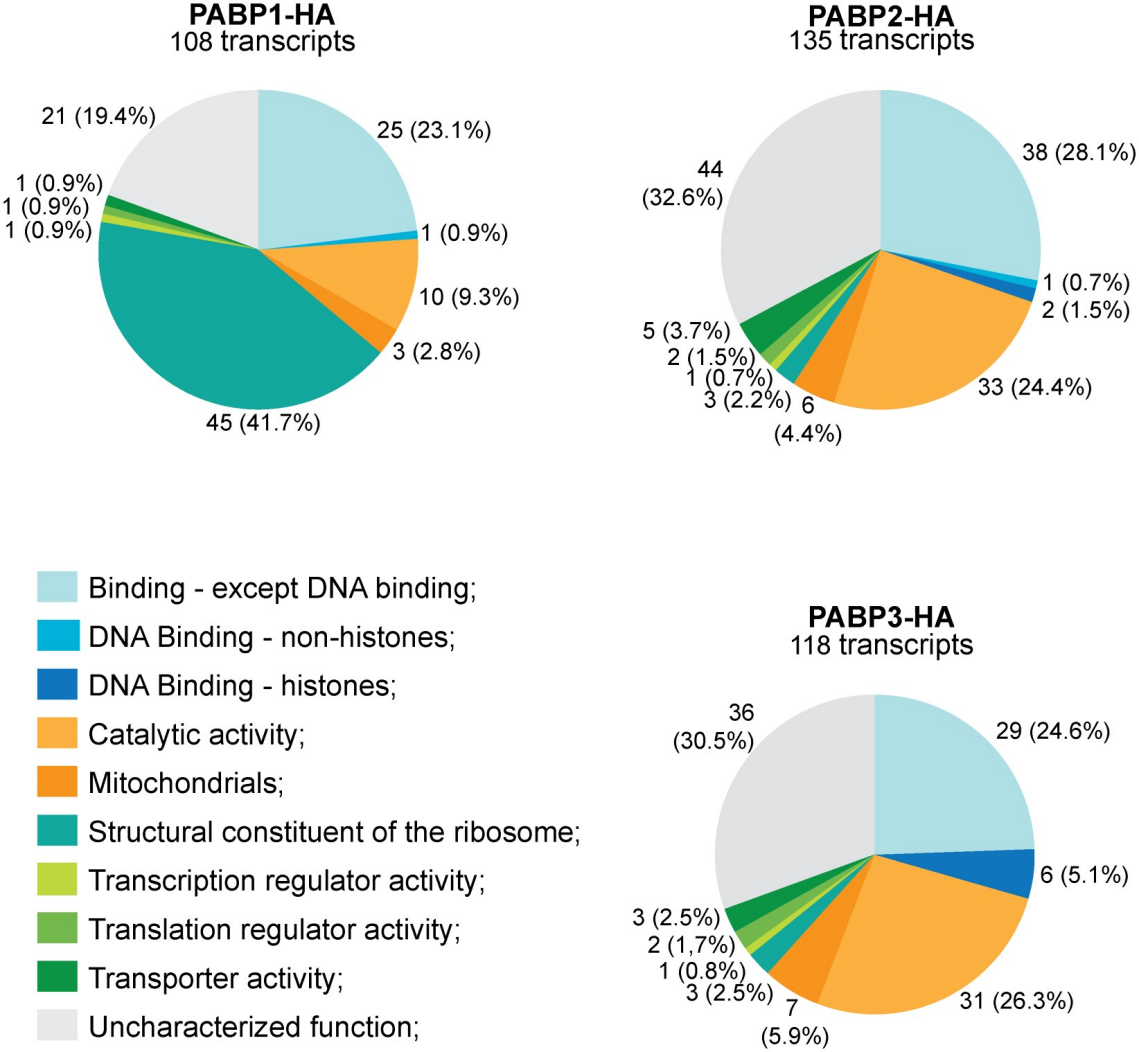
Analysis of mRNA populations associated with the three HA-tagged *Leishmania infantum* PABPs. mRNA groups associated with the recombinant HA-tagged PABP1, PABP2 and PABP3 from *L*. *infantum* (Illumina sequencing). Enriched genes in at least two of three available RNA-seq datasets were manually classified and grouped using the gene ontology (GO) terms according to their molecular function. ‘Enriched’ means at least 2-fold more abundant than in the negative control.

The full set of mRNAs identified with the new IP approach are listed in the S2 Table. A first analysis using a Volcano plot confirms a much-reduced overlap in mRNAs bound by PABP2 or PABP3 vs. PABP1. In contrast, no significant differences between the mRNAs associated with PABP2 or PABP3 can be detected (S3 Fig). A total of 108 mRNAs were found to co-precipitate with PABP1, fulfilling the criteria of log_2_ fold enrichment greater than two (Fig 1). Forty-five of the enriched transcripts (~42%) are mRNAs encoding ribosomal proteins, 10 (~9%) encode enzymes with “catalytic activity” and 26 (24%) encode proteins classified with the term “binding function”. PABP2 and PABP3 co-precipitated a total of 135 and 118 transcripts, respectively. For both PABPs, mRNAs encoding ribosomal proteins represented only ~2% of the co-precipitated transcripts, while 24% to 26% of those encode proteins with “catalytic activity” and roughly 30% encode proteins classified with “binding function”. Other GO functional categories were generally poorly represented among the mRNAs found associated with the three proteins. Noteworthy however, is the increased presence of histone mRNAs associated with PABP3 (six different transcripts–~5%) in comparison with both PABP2 (two transcripts– 1.5%) and PABP1 (one transcript—<1%), although the mRNAs co-precipitated with the HA-tagged *L*. *infantum* PABPs are not as enriched with histone transcripts as those co-precipitated with their native *L*. *major* orthologues (see S1 Table).

To better define the mRNAs specifically bound by individual HA-tagged PABPs and also to identify those commonly bound by distinct PABP pairs, we did a Venn diagram analysis of the transcripts co-precipitated with the three HA-tagged *L*. *infantum* PABPs (S4 Fig). A total of 66 mRNAs bound exclusively to PABP1, with 43 of those encoding ribosomal proteins. Meanwhile, 59 mRNAs bound exclusively to PABP2 and 37 to PABP3. PABP2 was associated with a larger number of mRNAs encoding hypothetical or uncharacterized proteins while the histone mRNAs were preferentially associated to PABP3. As expected, PAPB2 and PABP3 shared more mRNAs (45 total) than PABP1 and PABP2 (eight) or PABP1 and PABP3 (eleven). Twenty-three mRNAs, however, co-precipitated with all three proteins. Overall, the experiment with the HA-tagged proteins proved to be more specific than the one with the native proteins, with PABP1 having a clear preference for mRNAs encoding ribosomal proteins and those being noticeably absent from the transcripts associated with PABP2 or PABP3.

### *Leishmania* PABPs co-immunoprecipitate and interact with distinct RNA-binding proteins

Aside from their strong specificity for poly(A) sequences, PABPs have no known sequence specific affinity which could justify their selective binding to different sets of mRNAs. Binding to poly(A) has been shown to be mediated by specific residues within the PABPs’ RRMs 1 and 2, which are generally conserved in *Leishmania* PABP1 and PABP3, whereas substitutions in these residues have been identified for PABP2 which might lead to changes in sequence binding specificity [30]. For *Leishmania* PABP1 at least, and most likely for the PABP2/PABP3 pair as well, their recognition of specific mRNAs targets might require the assistance of partner RBPs with distinct RNA binding specificities. Here, putative RNA binding proteins that might be involved in specific mRNA recognition were initially identified in a pilot mass spectrometry analysis of the proteins co-immunoprecipitated with the three HA-tagged *L*. *infantum* PABPs (S5 Fig). These experiments led to the identification of RNA-binding proteins specifically associated with PABP1 (RBP23 and the uncharacterized LINF_180008000), PABP2 (DRBD2) or the three proteins (ZC3H41). Here we opted to investigate further both RBP23, confirmed to be strictly associated with PABP1 in both *Leishmania* and *T*. *brucei* [32,33], and DRBD2.

**Fig 2:**
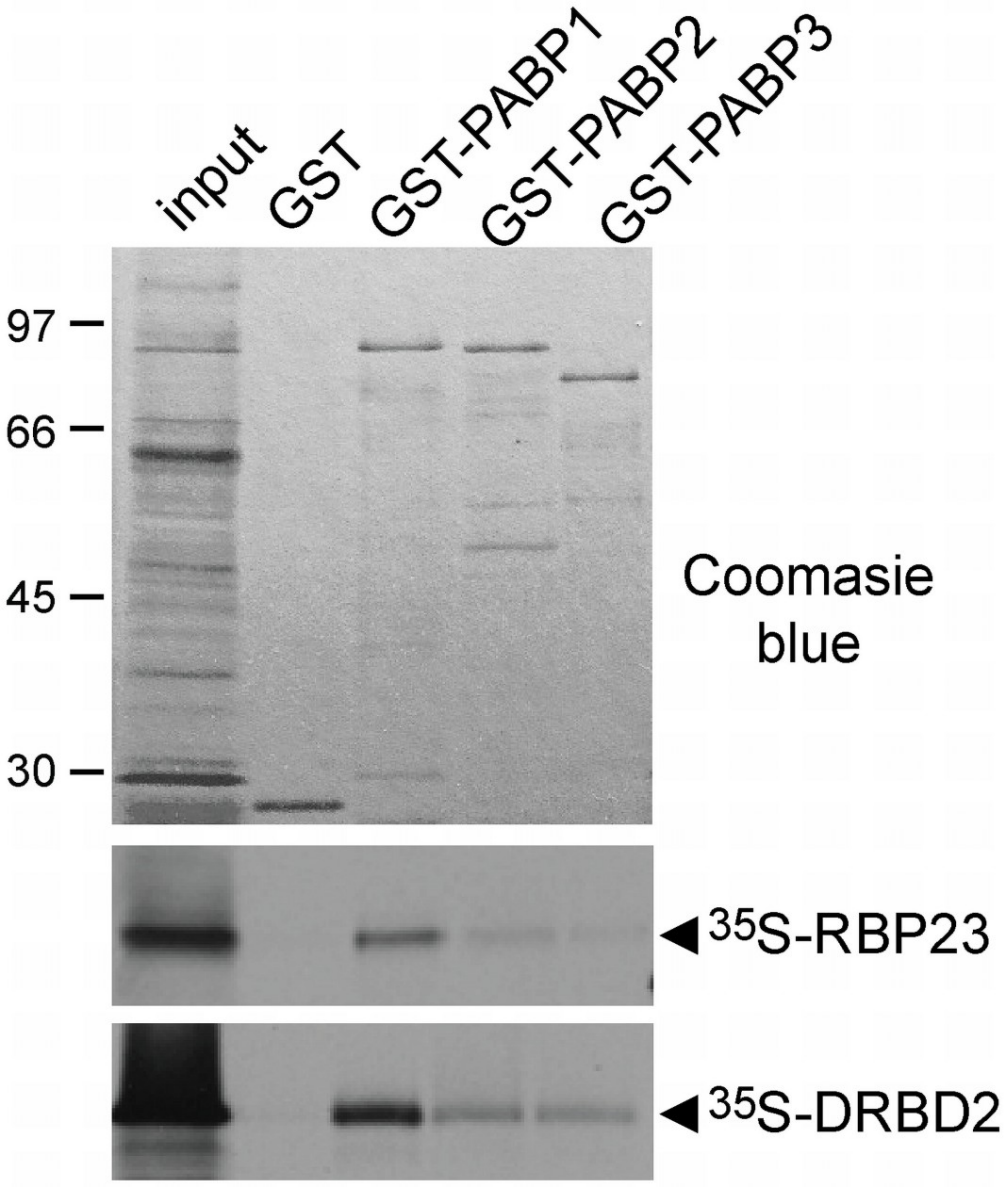
Protein-protein interaction assays investigating the direct binding between the *L*. *infantum* PABPs and their putative RBP23 and DRBD2 partners. Pull down assays between radiolabeled RBP23 or DRBD2 and three recombinant PABPs. The upper panel shows the Coomassie-blue stained gel indicating the radiolabeled protein input, the GST recombinant (negative control) and the GST-PABP homologues. The panels below are autoradiographs showing the result of the interactions between radiolabeled RBP23 or DRBD2 and recombinant proteins. The RBP23 and DRBD2 recombinant proteins are indicated by arrows.

In *T*. *brucei*, RBP23 is a cytoplasmic protein with a reticulated distribution, while DRBD2 was also localized within the cytoplasm, but distributed in the perinuclear region [39]. Both are small proteins characterized by the presence of RRM domains at their extremities, with the C-terminal RBP23 RRM and the two DRBD2 domains already mapped when these two proteins were originally described [40]. A second atypical RRM-like domain is also identifiable for RBP23 using current secondary structure and domain prediction tools, mapped to its N-terminus. To first confirm if direct interactions occur between RBP23 or DRBD2 and the *Leishmania* PABP homologues, both *RBP23* and *DRBD2* genes were cloned and the corresponding proteins used for *in vitro* pull-down assays. The three PABPs were first expressed in *Escherichia coli* with an N-terminal Glutathione S-transferase (GST) tag. After immobilization in Glutathione Sepharose these were then incubated with ^35^S-labeled RBP23 or DRBD2, produced by *in vitro* transcription/translation. The two ^35^S-labeled proteins bound to all three GST-tagged PABPs, with no binding seen for the negative GST control (Fig 2). However, a stronger signal was observed for both labeled proteins with PABP1, presumably indicating a stronger interaction. These results confirm the ability of both RBP23 and DRBD2 to directly interact with the *Leishmania* PABPs, with their binding motifs likely being conserved within different PABP sequences. Nevertheless, the lack of specificity seen *in vivo* by the co-immunoprecipitation assays, not only between RBP23 and PABP1 but also between DRBD2 and PABP2, indicates that this specificity might require binding to their mRNA targets or other protein partners.

### RBP23 binding partners

**Fig 3:**
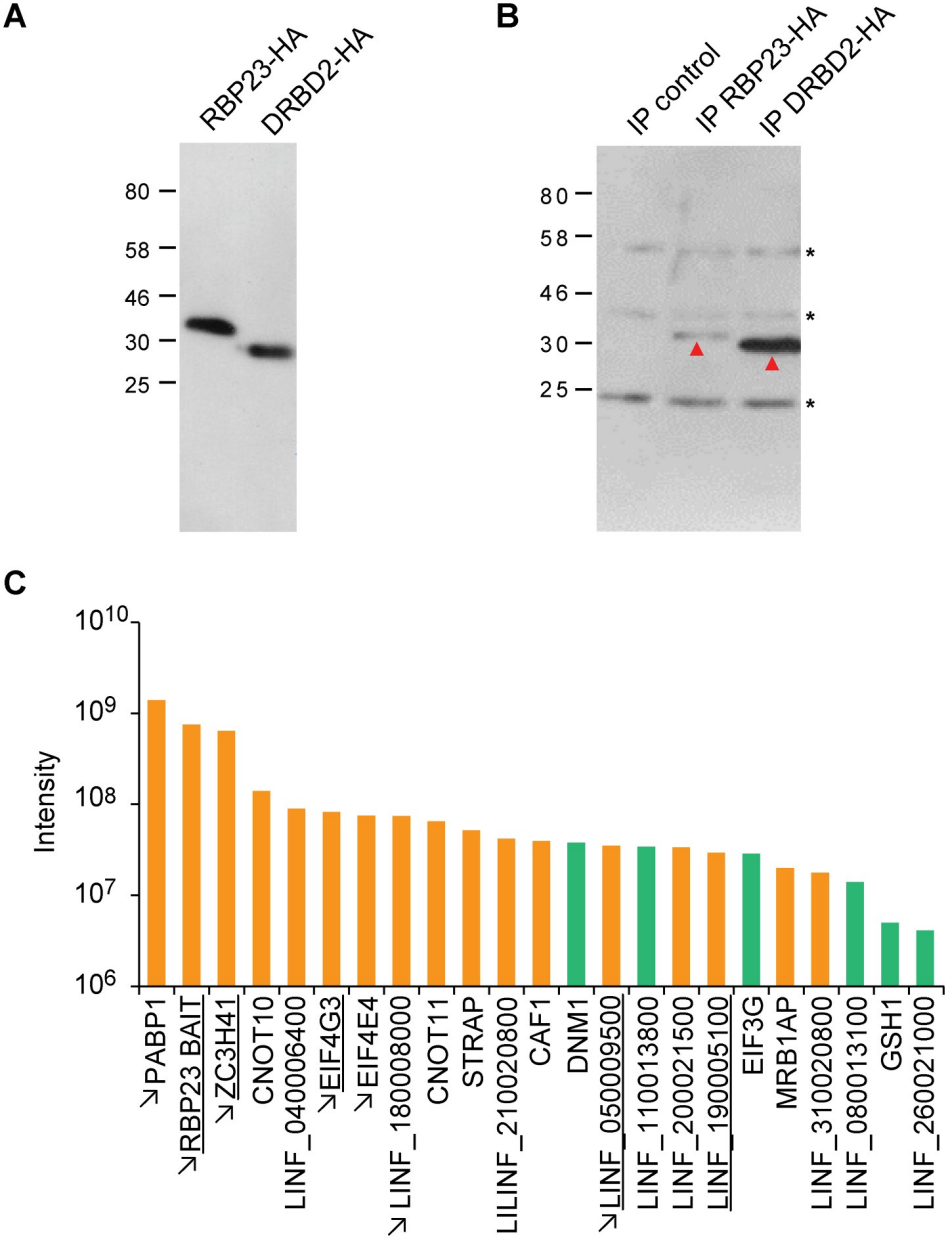
Expression of HA-tagged RBP23 and DRBD2 proteins and the top- co-purifying partners with RBP23. (**A**) Immunodetection of RBP23-HA or DRBD2-HA with monoclonal anti-HA antibody in whole *L*. *infantum* cell extracts expressing RBP23-HA or DRBD2-HA. (**B**) Immunodetection of the two HA-tagged proteins after immunoprecipitations with anti-HA beads using cytoplasmic extracts from the same *L*. *infantum* lines as well as from cells lacking HA-tagged proteins (IP control). The red arrows highlight the immunoprecipitated proteins, with the asterisks indicating IgG related bands. (**C**) Bar chart with proteins co-precipitated with RBP23 and having an enrichment strength defined as ‘+++’ (orange) and ‘++’ (green), according to Table 2. Proteins co-precipitated with both *L*. *infantum* HA-tagged PABP1 [33] and RBP23 are indicated by arrows. Proteins found to co-precipitate also with DRBD2 are underlined.

To fully investigate the RBP23 and DRBD2 interactions *in vivo*, both proteins were ectopically expressed in *L*. *infantum* with a C-terminal HA-tag. As for the PABPs, expression was confirmed with the anti-HA antibody in exponentially grown promastigotes, with both proteins migrating as single bands in agreement with predicted sizes (Fig 3A). No isoforms suggestive of post-translational modifications were seen. Cytoplasmic extracts were then generated, and preliminary IPs performed. The amount of RBP23 detected in the cytoplasmic extract and/or IPs was generally much lower than that observed for DRBD2 (Fig 3B), contrasting with equivalent levels of expression for both proteins in whole cell extracts. RBP23 is thus likely to be much more susceptible to degradation than DRBD2.

To confirm the interaction with PABP1 and to identify additional protein partners of functional relevance, proteins co-immunoprecipitated with the HA-tagged RBP23 were next submitted to mass-spectrometry. For these assays we used extracts that were not RNase treated so that both directly bound partners as well as proteins bound to the same mRNAs as RBP23 would be precipitated. Normalized intensities from two sets of replicates, and with a minimum of two peptides found for each replicate, were first used to generate a full list of proteins enriched 1.5-fold or more with RBP23 in comparison to the negative control (S3 Table). For a more detailed analysis, we first considered only co-precipitated proteins that were enriched by four-fold or more in comparison with the control. These were categorized, based on both the intensity values and the ratio of enrichment, according to the “strength” of association with RBP23 (Tables 1 and S4). Proteins classified within the top two categories, possibly reflecting more robust associations, are summarized in Fig 3C. PABP1 was found to be foremost among those, being the top-most protein in intensity and with a greater than 65-fold enrichment. In contrast, although PABP2 can be considered high ranking among the co-precipitated proteins listed in the S3 Table, due to its overall intensity value, it is only enriched between three- to four-fold, similarly to PABP3. EIF4G3 and EIF4E4 are also among the top-most proteins enriched with RBP23, as well as three other proteins previously shown to specifically co-precipitate with the *L*. *infantum* HA-tagged PABP1 [33]: the zinc-finger ZC3H41; the uncharacterized LINF_180008000; and LINF_050009500, another uncharacterized protein structurally similar to Skp1 (S-phase kinase associated protein 1).

**Table 1:** Proteins *specifically* co-immunoprecipitating with *L*. *infantum* RBP23.

TRITrypDB ID	Name	Description	Intensity	Ratio	Strength
**Bait**					
**→**LINF_170011800	RBP23	RNA-binding protein 23	7.6x10^8^	291.1	+++
**Translation initiation factors and partners**					
**→**LINF_350055900	PABP1	Poly(A)-binding protein 1	1.4x10^9^	66	+++
**→** LINF_160022100	EIF4G3	Eukaryotic translation initiation factor 4G3	8.3x10^7^	25.4	+++
**→**LINF_300009600	EIF4E4	Eukaryotic translation initiation factor 4E4	7.5x10^7^	129.8	+++
LINF_340033000	EIF3G	Eukaryotic translation initiation factor 3 G	2.9x10^7^	4.3	++
LINF_330010200	4E1-IP2	EIF4E1-interacting protein 2	2.1x10^6^	6.5	-
LINF_270023100	EIF4E1	Eukaryotic translation initiation factor 4E1	1.7x10^6^	4.8	-
**RNA-binding proteins and related**					
**→** LINF_270019700	ZC3H41	CCCH-Zinc finger 41	6.5x10^8^	10.1	+++
LINF_270018900	STRAP	Serine-threonine kinase receptor-associated protein	5.2x10^7^	6.1	+++
LINF_110015300	MRB1AP	Mitochondrial-associated protein	2x10^7^	36.8	+++
LINF_300016700	TRRM2	Three RRMs containing protein 2	6.8x10^6^	7.8	-
LINF_310020800	PuREBP1	Purine-Responsive Element Binding Protein 1	1.8x10^7^	13.7	+++
**Polyadenylation and Deadenylation**					
LINF_360070900	CNOT10	CCR4-NOT complex subunit 10	1.4x10^8^	7.9	+++
LINF_230005100	CNOT11	CCR4-NOT complex subunit 11	6.5x10^7^	7.3	+++
LINF_220022600	CAF1	CCR4-associated factor	4x10^7^	10.7	+++
LINF_240005900	PAP	Poly A polymerase, regulatory subunit	3.3x10^6^	11.8	-
LINF_100018400	PBP1	PAB1-binding protein	2.3x10^6^	5.6	-
**Enzymes**					
LINF_180022300	GSH1	Gamma-glutamylcysteine synthetase	5x10^6^	11	++
LINF_060014300	DHFR-TS	Dihydrofolate reductase-thymidylate synthase	3x10^6^	5.9	-
LINF_300036300		tRNA (Guanine-1)-methyltransferase	7.6x10^5^	5.2	-
**Intracellular transport and cell motility**					
LINF_290029700	DNM1	Dynamin-1-like protein	3.8x10^7^	5	++
**Uncharacterized proteins with identifiable domains**					
→LINF_180008000		Protein with NFT2 and RRM domains	7.4x10^7^	23.9	+++
LINF_210020800		PPR domain protein	4.2x10^7^	16.4	+++
→LINF_050009500	Skp1-like	Skp1 (S-phase kinase-associated protein 1)—like	3.5x10^7^	10.6	+++
LINF_080013100		BAR domain protein	1.4x10^7^	11.7	++
LINF_260021000		RING-zinc finger protein	4.1x10^6^	12.2	++
**Other selected uncharacterized proteins**					
LINF_040006400			9x10^7^	15.6	+++
LINF_110013800			3.4x10^7^	4.1	++
LINF_200021500			3.4x10^7^	81.4	+++
LINF_190005100			2.9x10^7^	10.3	+++

The polypeptides listed are also detailed in the S3 Table and includes only identified proteins which fit into the following parameters: two or more peptides found for each *RBP23* replicate; a minimum of 4-fold (4x) enrichment over the negative control (“Ratio”); a minimum average intensity (“Intensity”) 1000-fold (1000x) less than that seen for the RBP23 bait. The “Strength” column defines the strength of association with RBP23 taken into account both the average intensity and the enrichment in comparison with the negative control, according with criteria defined in the bottom of the S3 Table. Proteins co-immunoprecipitated with both L. infantum HA-tagged PABP1 [33] and RBP23 are indicated by arrows. Proteins also found co-precipitated with DRBD2 are underlined.

Several proteins among those shortlisted in Table 1 and Fig 3C have specific functions associated with RNA metabolism. Noteworthy, are the CNOT10, CNOT11 and CAF1 subunits of the CCR4-NOT complex. Two proteins linked with mitochondrial mRNA metabolism, PPR and MRB1, were also found, as well as STRAP, an RNA-binding protein whose *T*. *brucei* orthologue localizes to stress granules under starvation conditions [41]. Uncharacterized co-precipitated proteins whose *T*. *brucei* orthologues have both been shown to be associated with mRNA include LINF_200021500 and LINF_190005100 [42]. The LINF_200021500 orthologue was also found to enhance expression in a tethering assay, and seen to bind to MKT1, a known translational regulator [43,44]. Another uncharacterized protein whose orthologue was also found to stimulate expression is LINF_110013800. Considering that in *T*. *brucei* the PABP1, EIF4E4, EIF4G3, EIF4AI and LINF_180008000 orthologues all also enhance expression, with some at least through translation stimulation, the strong association seen with these proteins reinforce a possible role for RBP23 during translation initiation.

### DRBD2 binding partners

**Fig 4:**
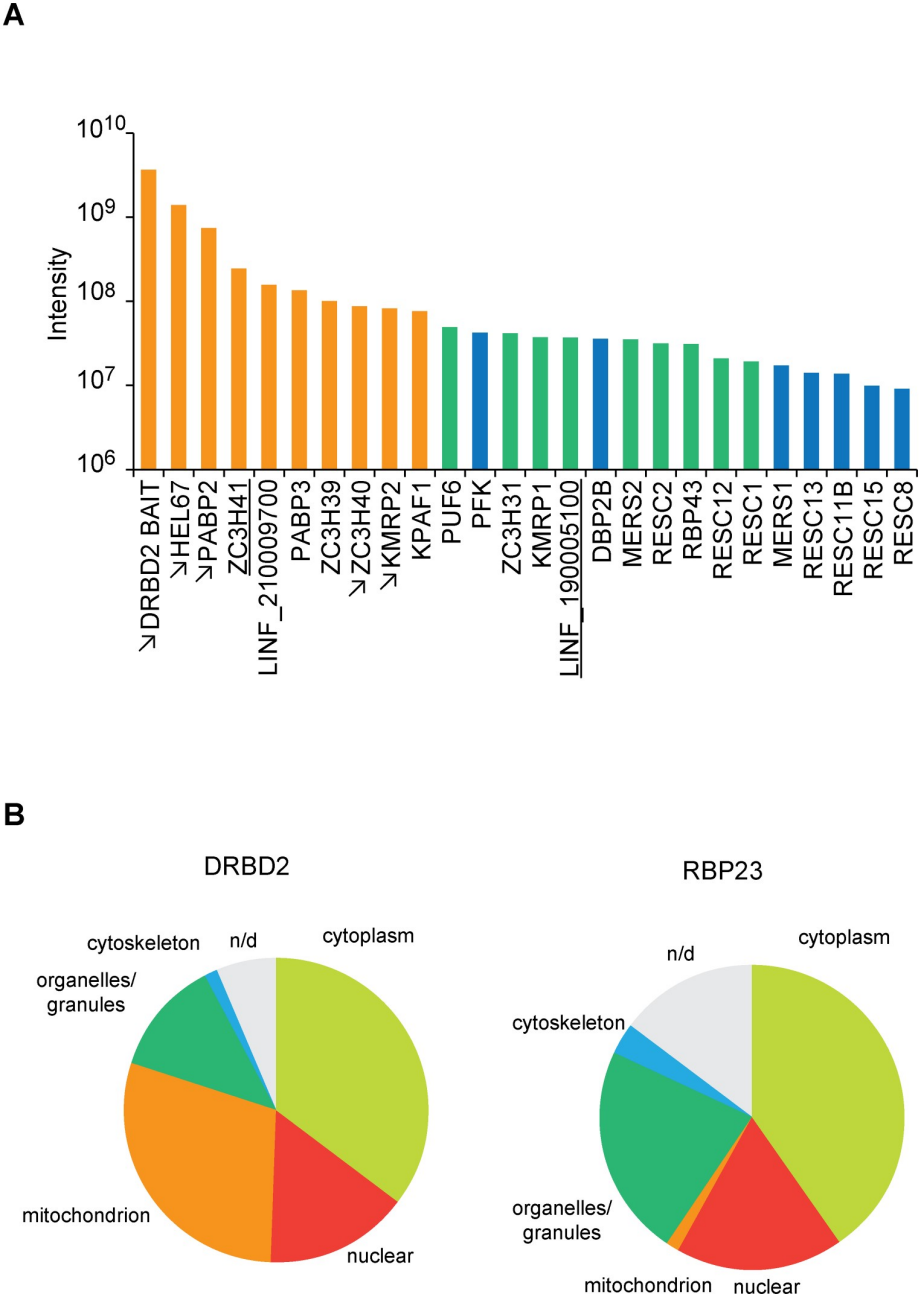
Top proteins co-purifying with HA-tagged DRBD2 and their subcellular compartmental localization compared to HA-tagged RBP23. (**A**) Bar chart with proteins co-precipitated with DRBD2 with the enrichment strength defined as ‘+++’ (orange), ‘++’ (green) and ‘+’ (blue), according to Table 2. Proteins co-purifying with both *T. cruzi* DRBD2 [45] and its *L. infantum* HA-tagged orthologue are indicated by arrows. Proteins in common with RBP23 are underlined. (**B**) Pie charts with subcellular localization of DRBD2 (left chart) or RBP23 (right chart) partners. This was defined based on cellular component classes: cytoskeleton; cytoplasm; mitochondrion; nuclear; organelles/granules (except mitochondria); n/d or not defined. For uncharacterized proteins, their localization was defined based on their respective orthologues from *T*. *brucei* and according to TrypTag.org.

Results derived from a mass-spectrometry analysis of DRBD2-HA samples were analysed generally as described for RBP23, with the full list of enriched proteins shown in the S5 Table. Proteins enriched 4-fold or more and categorized as to the “strength” of association with DRBD2 are listed in Table 2 and S6 Table and the top-most are also represented in Fig 4A. The data confirm that DRBD2 preferentially co-precipitates not only with PABP2 but also with PABP3. It furthers highlights the absence of PABP1, or most other proteins associated with RBP23, from the topmost co-precipitated proteins. Although RBP23 is found in the list from Table 2, it is one of the lowest ranking proteins included there. Only two proteins were found among the top candidates co-precipitated with both RBP23 and DRBD2: ZC3H41 and the uncharacterized LINF_190005100. Several RNA-binding proteins, not generally seen with RBP23, were found in the DRBD2 pull-down, contrasting with a reduced number of uncharacterized proteins. Considering only those from the top two categories, and omitting mitochondrial proteins, a total of four proteins with a zinc-finger domain (ZC3H31, ZCH39, ZCH40 and ZCH41), one RRM containing RBP (RBP43), a pumilio domain protein (PUF6), a putative RBP with NTF2 domain (LINF_210009700) and one DEAD-box RNA helicase (HEL67/DDX3/Ded1p) were found in our analyses.

**Table 2:** Proteins *specifically* co-immunoprecipitating with *L*. *infantum* DRBD2.

TriTrypDB ID	Name	Description	Intensity	Ratio	Strength
**Bait**					
**→**LINF_350027200	DRBD2	dsRNA-binding domain 2	3.7x10^8^	110.3	+++
**Translation initiation factors**					
**→**LINF_350047100	PABP2	Poly(A)-binding protein 2	7.4x10^8^	12.4	+++
LINF_250005800	PABP3	Poly(A)-binding protein 3	1.3x10^8^	13.9	+++
LINF_160022100	EIF4G3	Eukaryotic translation initiation factor 4G3	1.9x10^7^	4.4	-
**RNA-binding proteins**					
LINF_270019700	ZC3H41	CCCH-Zinc finger 41	2.5x10^8^	4.7	+++
LINF_210009700		RNA-binding protein with NTF2 domain	1.6x10^8^	13.1	+++
LINF_190007800	ZC3H39	CCCH-Zinc finger 39	1x10^8^	63.3	+++
**→**LINF_190007900	ZC3H40	CCCH-Zinc finger 40	8.8x10^7^	44.4	+++
LINF_330019100	PUF6	Pumilio protein 6	4.9x10^7^	15.6	++
LINF_360013000	ZC3H31	CCCH-Zinc finger 31	4.2x10^7^	11	++
LINF_250008000	RBP43/ G1-IP2	RNA-binding protein 43	3.1x10^7^	13.6	++
LINF_180010900	RBP12	RNA-binding protein 12	2.7x10^7^	7.1	-
LINF_270028100	TRRM1	Three RRMs containing protein 1	1.5x10^7^	4.8	-
LINF_300016700	TRRM2	Three RRMs containing protein 2	1.3x10^7^	4.7	-
LINF_320012700	PAN3	Poly(A) Specific Ribonuclease Subunit	7.1x10^6^	6	-
LINF_170011800	RBP23	RNA-binding protein 23	6.5x10^6^	4.8	-
Mitochondrial RNA-binding proteins*					
**→**LINF_090018500	KMRP2/ MRP2	Mitochondrial RNA binding protein 2	8.2x10^7^	16.5	+++
LINF_180005000	KPAF1	Kinetoplast polyadenylation factor 1	7.7x10^7^	38.8	+++
LINF_270017500	KMRP1/ MRP1	Mitochondrial RNA binding protein 1	3.8x10^7^	11.7	++
LINF_280005300	MERS2	Mitochondrial edited mRNA stability factor 2	3.5x10^7^	34.5	++
LINF_220011900	RESC2/ GAP2	Guide RNA associated protein 2	3.2x10^7^	44.3	++
LINF_310012100	RESC12/ REMC5	RNA editing mediator complex protein 5	2.1x10^7^	14.2	++
LINF_330036600	RESC1/ GAP1	Guide RNA associated protein 1	1.9x10^7^	22.4	++
LINF_320031100	MERS1	Mitochondrial edited mRNA stability factor 1	1.7x10^7^	100.8	+
LINF_080016600	RESC6/ GRBC6	gRNA binding complex subunit 6	1.5x10^7^	8.3	-
LINF_330007800	RESC13/ RGG2	RGG-containing protein 2	1.4x10^7^	26.1	+
LINF_310012000	RESC11B/ MRB4150	Mitochondrial RNA binding complex 1 subunit	1.4x10^7^	12.9	+
LINF_200014400	RESC15/ PAMC1	Polyadenylation mediator complex 1	9.9x10^6^	11.8	+
LINF_360057200	RESC8/ REMC1	RNA editing mediator complex protein 1	9.18x10^6^	16.8	+
LINF_330020200	RESC5/ GRBC5	gRNA binding complex subunit 5	8.8x10^6^	15.5	-
LINF_300013200	KRGG1/ RGG1	RGG-containing protein 1	6.2x10^6^	11.3	-
LINF_210011600	RESC17/ PAMC3	Polyadenylation mediator complex 3	5.8x10^6^	8.3	-
LINF_250021900	KRBP72/ MRB1590	Mitochondrial RNA binding complex 1 subunit	4.6x10^6^	4.2	-
LINF_360078000	RESC19/ MERS3	Mitochondrial edited mRNA stability factor 3	3.8x10^6^	32.7	-
**RNA helicases**					
**→**LINF_320009100	HEL67	ATP-dependent RNA helicase	1.4x10^9^	7.7	+++
LINF_070008800	DBP2B	ATP-dependent DEAD/H RNA helicase	3.6x10^7^	4.9	+
LINF_050006300	DDX21	Nucleolar RNA helicase II	2.2x10^7^	7	-
LINF_220021200		ATP-dependent DEAD/H RNA helicase	4.6x10^6^	4.2	-
**RNA methyltransferases**					
LINF_110010500	G1-IP	mRNA cap guanine-N7 methyltransferase	3.4x10^7^	6.2	-
LINF_200021600		tRNA (Uracil-5-)-methyltransferase	4.3x10^6^	7.4	-
**Other enzymes**					
LINF_290032900	PFK	ATP-dependent phosphofructokinase	4.2x10^7^	7.6	+
LINF_300039500	PAS-PGK	PAS-domain containing phosphoglycerate kinase	5x10^6^	7.1	-
LINF_060018400	MO	Monooxygenase	4.3x10^6^	4.6	-
LINF_280021800	ABH	Hydrolase—alpha/beta fold family	3.9x10^6^	7.8	-
LINF_190022000	IMPDH	Inosine-5’-monophosphate dehydrogenase	3.6x10^6^	9.2	-
**Uncharacterized proteins with other identifiable domains**					
→LINF_050009500	Skp1-like	S-phase kinase-associated protein 1—like	1.4x10^7^	4.4	-
**Uncharacterized proteins**					
LINF_190005100			3.7x10^7^	31.4	++
LINF_200005800			6.2x10^6^	7.1	-

The polypeptides listed here are detailed in S5 Table and include only identified proteins which fit into the following parameters: two or more peptides found for each DRBD2 replicate; a minimum of 4-fold (4x) enrichment over the negative control (“Ratio”); a minimum average intensity (“Intensity”) 1000-fold (1000x) less than that seen for the DRBD2 bait. The “Strength” column defines the strength of association with DRBD2 taking into account both the average intensity and the enrichment in comparison to the negative control, according to the criteria defined in the bottom of the S5 Table. Proteins co-purifying with both *T*. *cruzi* DRBD2 [45] and *L*. *infantum* HA-tagged DRBD2 are indicated by arrows. Proteins in common with RBP23 are underlined.

*include recently assigned names [46].

A relevant observation from the DRBD2 data is the co-precipitation of 18 proteins with defined functions associated with mitochondrial RNA (Table 2), most of which showing substantial enrichments (greater than 15-fold). The mitochondrial-related proteins represent 38% of those found associated with DRBD2, as opposed to the putative RBP23 partners, of which only 2% belong to this category (Fig 4B). Orthologues to several of the proteins co-precipitated with the *Leishmania* DRBD2, but none of the mitochondrial proteins, were also found in a recent analysis of DRBD2 binding partners from *T*. *cruzi* [45]. Overall, the set of proteins found associated with DRBD2 highlights a markedly distinct profile in comparison with RBP23. It also suggests possible involvement in many different processes associated with the metabolism of targeted mRNAs but with minor, if any, role in translation.

### Comparative analysis of selected proteins co-immunoprecipitated with RBP23 and DRBD2

**Fig 5:**
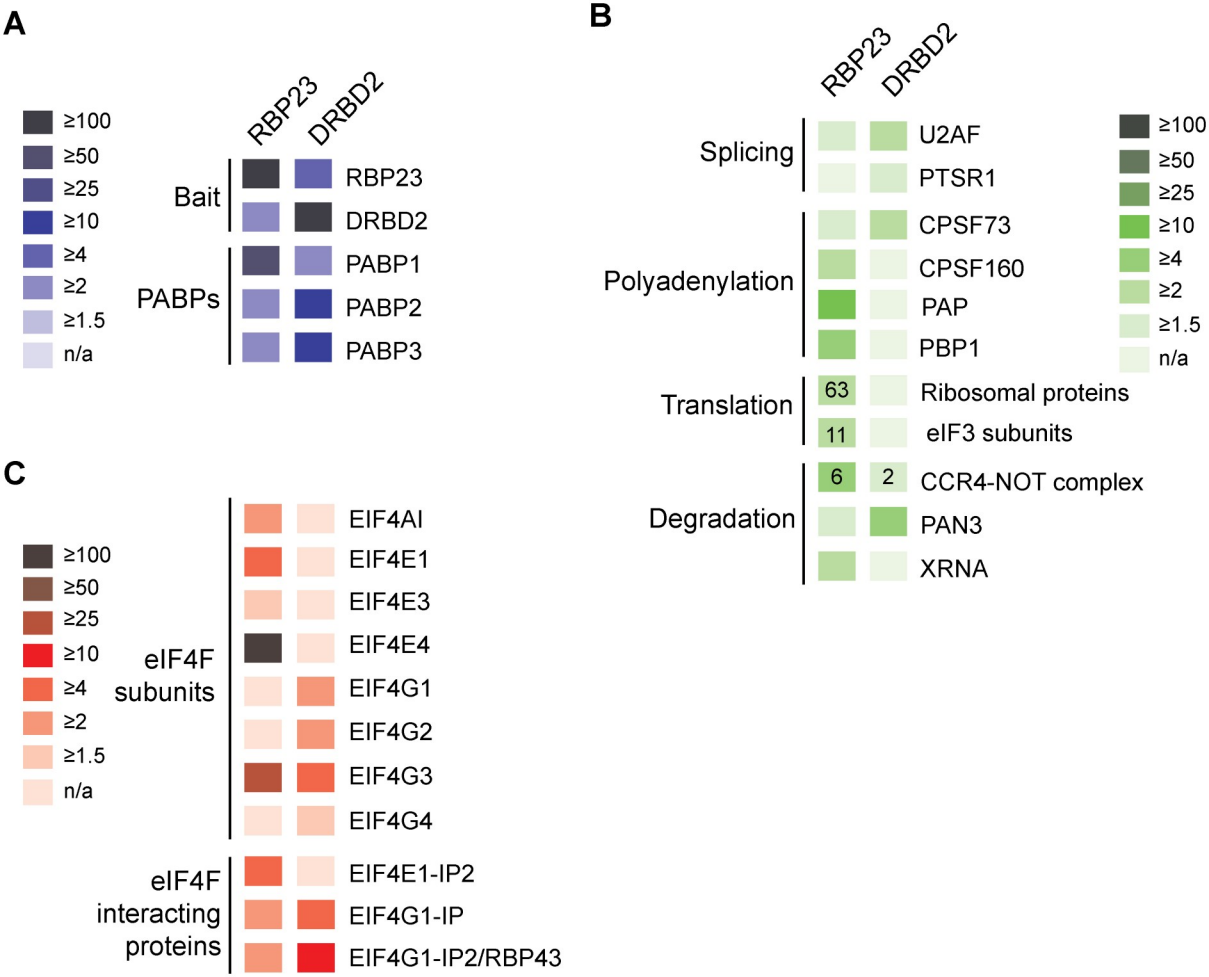
Comparative enrichment ratios for the major protein groups co-precipitated with *L*. *infantum* HA-tagged RBP23 and DRBD2. Proteins co-precipitated with RBP23 and DRBD2 belonging to the selected functional categories related to mRNA processes are represented by boxes coloured according to the enrichment ratios derived from S3 and S5 Tables. The numbers inside the boxes indicate the number of subunits of the named complex (including ribosomes) that have equivalent enrichment ratios. (**A**) Enrichment ratios for the baits and three PABP homologues. (**B**) Enrichment ratios for eIF4F subunits and eIF4F interacting proteins. (**C**) Enrichment ratios for other proteins with functions related to mRNA splicing, polyadenylation, translation or degradation.

To better define the association of RBP23 or DRBD2 with different co-immunoprecipitated putative partners, we directly compared the enrichment ratios seen with either RBP23 or DRBD2 for several proteins known to be specifically associated with major RNA processes. First, as shown in Fig 5A, the comparison clearly highlights the association of RBP23 with PABP1, while DRBD2 preferentially interacts with PABP2 and also co-precipitates with PABP3. A differential association was also seen with a number of proteins and enzymes involved with different aspects of RNA metabolism (Fig 5B), such as various splicing and polyadenylation factors, some enriched only with RBP23 and others with DRBD2. In contrast, enrichments for over 60 ribosomal protein subunits and all 11 experimentally validated eIF3 subunits [47] were found with RBP23, but not with DRBD2. These results are consistent with RBP23 being associated with polysomal mRNAs, in contrast to DRBD2, and also in agreement with published tethered results showing that RBP23 enhances expression while DRBD2 has been shown to repress expression [42,43]. As previously, highlighted, another contrasting difference between the two proteins is the association of RBP23 with six subunits of the CCR4-Not complex, some with a very substantial enrichment, not seen for DRBD2.

Here we also sought to investigate in more detail the association of both RBP23 and DRBD2 with eIF4F subunits and related proteins. As well as the EIF4E4/EIF4G3 complex, EIF4AI, its known helicase partner in trypanosomatids, was differentially co-precipitated with RBP23 only (Fig 5C). EIF4E1, another eIF4E homologue, and one of its known protein partners, EIF4E1-IP2, was also found enriched with RBP23. In contrast, two eIF4G homologues, EIF4G1, with partners EIF4G1-IP and EIF4G1-IP2 (also called RBP43), and EIF4G2, were more enriched with DRBD2, with EIF4G3 being enriched not only with RBP23 but also with DRBD2. Little or no enrichment was seen with either of the RBPs for EIF4E3, the most abundant of the trypanosomatid eIF4Es, and its EIF4G4 partner. Once again, these differences highlight the very distinct profile of protein partners co-precipitating with both RBPs.

### Distinct mRNA populations co-immunoprecipitated with RBP23 and DRBD2

**Fig 6:**
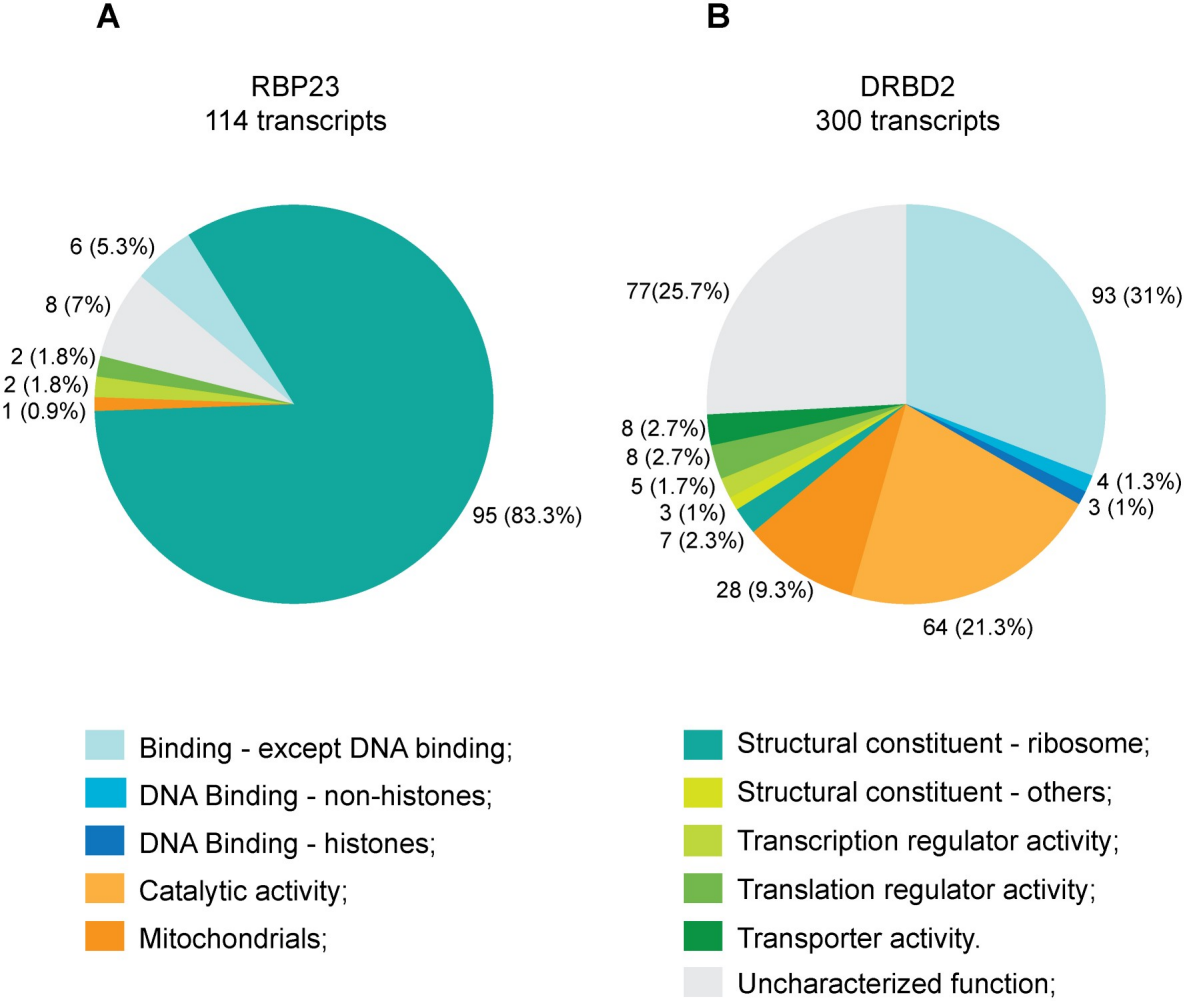
Analysis of mRNA populations associated with the HA-tagged RBP23 and DRBD2. (**A**) mRNA groups associated with RBP23. (**B**) mRNA groups associated with DRBD2. Transcripts were classified and grouped as detailed in Fig 1, with an extra category added to specifically highlight the mitochondrial proteins.

To define the putative mRNA targets bound by the either RBP23 or DRBD2 and to investigate overlaps and differences in comparison to their PABP partners, sequencing of RNA populations co-immunoprecipitated with HA-tagged RBP23 and DRBD2 was performed using the same procedures carried out with the HA-tagged PABPs. The full list of mRNAs specifically enriched with either RBP23 or DRBD2, according to the same criteria defined for the analysis with the mRNAs bound to the three PABPs, is found in the S7 Table. These mRNAs are also functionally grouped in Fig 6A. A total of 114 enriched mRNAs were co-precipitated with RBP23, of which the vast majority (>80%) encode known ribosomal proteins. RBP23 thus show a much greater specificity than that seen for either the native or HA-tagged PABP1. Among the remaining enriched transcripts, at least three are directed linked to ribosomal function: the translation factor EIF5A, the nuclear RNA binding protein NRBP and the nascent polypeptide associated complex subunit. Comparison of the mRNAs co-precipitated with the *L*. *infantum* PABP1 to those found associated with RBP23 revealed that ~90% of the ribosomal protein mRNAs found with PABP1 also co-precipitated with RBP23. In contrast, none of the mRNAs encoding uncharacterized proteins found with PABP1, the second most abundant category of mRNAs found with this protein, were among those co-precipitated with RBP23. A very interesting observation, however, is that the top-most mRNA enriched with RBP23 was its own transcript, not found among those associated with PABP1.

A remarkably different profile was observed for the mRNAs co-precipitated with DRBD2 (Fig 6B). From a total of 300 enriched transcripts, only a minor fraction of those (~2%) encode proteins with a structural activity role. Among the 14 transcripts that encode ribosomal protein mRNAs, half encode subunits of mitochondrial ribosomes, not found associated with RBP23 (classified under mitochondrial in Fig 6B). For the remaining transcripts, most can be classified into three categories: ~31% encode binding proteins, ~26% encode proteins with uncharacterized functions and ~21% encode enzymes with catalytic activity. When compared with the PABP2 or PABP3 co-precipitated transcripts, a very limited overlap was seen (<3%) in specific mRNAs co-precipitated with DRBD2. It is possible that the reduced overlap might reflect the much greater diversity of mRNAs, both in number and functional categories, bound by these proteins, with only a limited fraction of those being identifiable by the approach used. Nevertheless, a possible difference seen between DRBD2 and PABP2/PABP3 is a greater association with DRBD2 of mRNAs encoding mitochondrial proteins.

### *In silico* 3’UTR motifs within target mRNAs associated with the RBP23 or DRBD2 proteins

**Fig 7:**
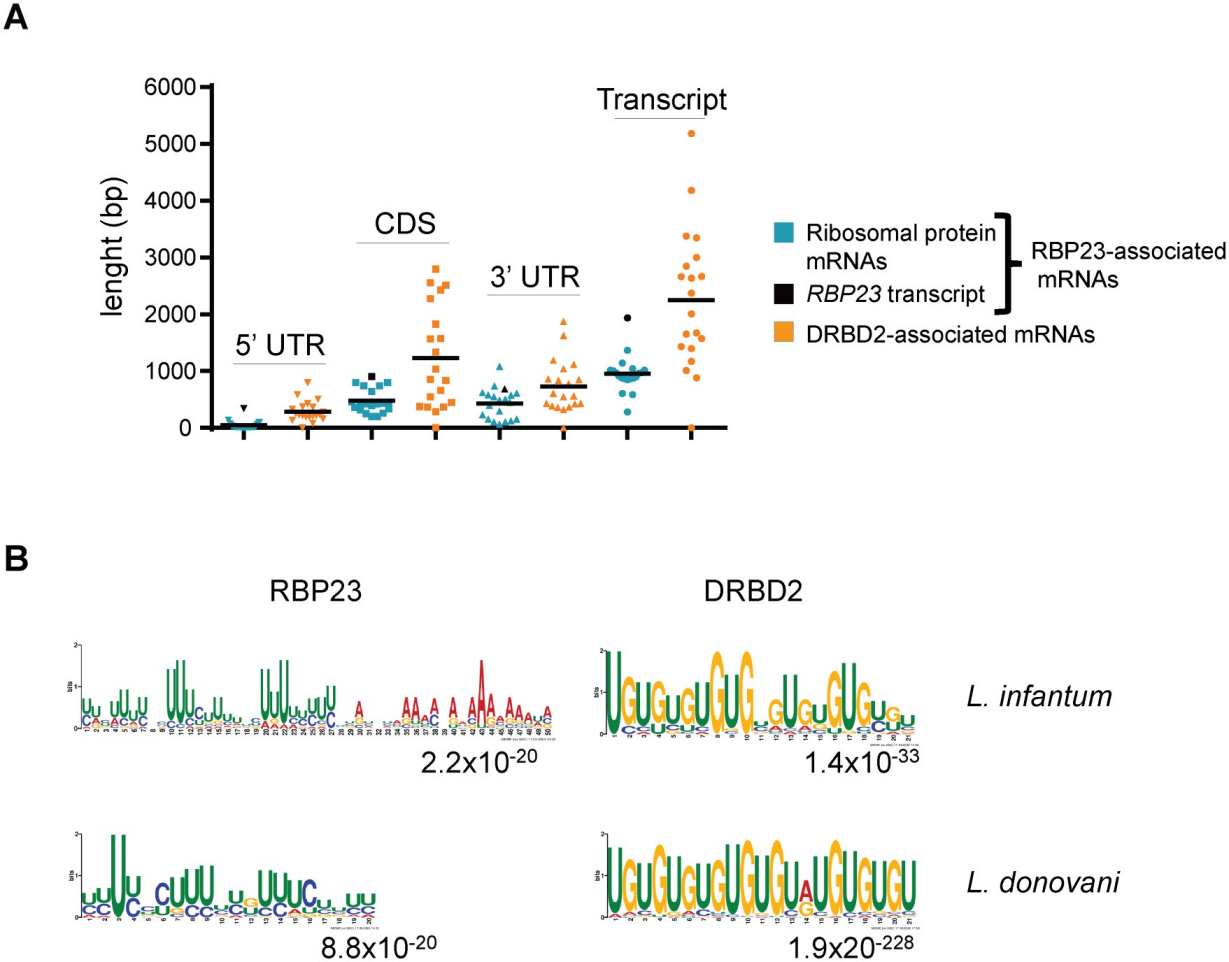
Sequence elements within the 3’UTRs of trypanosomatid mRNAs associated with RBP23 and DRBD2. (**A**) Comparison of RBP23 (blue) and DRBD2 (orange) 5’ (▼) and 3’ (▲) UTRs, CDS (■) and total transcript lengths (●) according to *L*. *donovani* orthologous sequences [48]. The mean values are represented by black lines and the *RBP23* transcript is indicated by the same symbols coloured in black. (**B**) Putative motifs found within 3’UTRs from the top 20 mRNAs associated to either HA-tagged RBP23 or DRBD2 in *L*. *infantum* and *L*. *donovani*. The motifs found in searches with mRNAs enriched with the HA-tagged RBP23, including the *RBP23* transcript as well as the top 19 mRNAs encoding ribosomal proteins, are shown on the left. Those motifs found for the top 20 mRNAs associated to HA-tagged DRBD2 are shown on the right. All logos were generated by MEME (*Multiple Em for Motif Elicitation*) and the relative height of each nucleotide represents the conservation at that specific position. The e-values are in the right corner of each logo.

In order to define 3’UTR sequence elements which could mediate any specific association between RBP23/DRBD2 and target mRNAs, we first used transcript length data available from *L*. *donovani* orthologues [48] to compare the length of coding sequence and untranslated regions of the top-most 20 transcripts enriched with either RBP23 or DRBD2 (Fig 7A). This comparison highlights noticeable differences in length between these mRNAs, with the RBP23-associated mRNAs having much shorter 5’UTRs and coding regions than the DRBD2-associated transcripts, as well as smaller 3’UTRs. With the sole exception of the *RBP23* transcript, the other top 19 transcripts co-precipitated with RBP23 encode ribosomal proteins and a remarkable feature of these mRNAs in trypanosomatids is their small 5’UTRs, as reported from *T*. *brucei* [49]. Based on the *L*. *donovani* transcriptome data, and after excluding the 39 nucleotides spliced leader (mini-exon) sequence, these can be as small as eight nucleotides in length [48]. As highlighted in Fig 7A, the native *RBP23* transcript differs from the top-most messages bound by the HA-tagged RBP23 in having a larger 5’UTR and coding region. mRNAs encoding both the native RBP23 and the ectopically expressed HA-tagged protein were specifically co-precipitated. This observation excludes any binding by RBP23 to the 5’ or 3’UTRs of its native mRNA, absent from the ectopically encoded protein. It implies either an association with the protein coding sequence or, alternatively, a co-translational dimerization between the tagged protein and the nascent polypeptide, leading to its co-precipitation with the attached mRNA. It also reinforces that the nature of the association between ribosomal protein mRNAs and RBP23 is most-likely distinct from that between RBP23 and its own transcript.

Next, we searched for any sequence elements within the same set of RBP23 or DRBD2 co-precipitated transcripts that would allow them to be specifically selected by either protein. Considering the much-reduced size for the 5’UTRs of the RBP23-associated mRNAs, the search focused on elements localized to their 3’UTRs using 300 nucleotides immediately downstream of the *L*. *infantum* coding sequences and, independently, the mapped 3’UTRs of the equivalent *L*. *donovani* transcripts. Motifs rich in uridines were found to be a common feature within the 3’UTRs of the RBP23 bound transcripts in both *L*. *infantum* and *L*. *donovani* sequences (Fig 7B). These motifs were found in one to four copies in most 3’UTRs of the ribosomal protein mRNAs tested that co-precipitated with RBP23 but were noticeably absent from the 3’UTR of the *RBP23* transcript (S6 Fig). Comparatively, for the 3’-UTRs of the mRNAs associated with DRBD2, a motif with UG repeats was found in both *Leishmania* species in nearly all mRNAs investigated (Figs 7B and S6). The markedly distinct motifs identified within the transcripts found associated with each protein indicate that, at least for the functionally related RBP23-bound messages, they might have a role in defining the association with these RBPs. It remains to be seen whether they would be directly recognized by these proteins or their recognition would require extra RNA-motifs and/or protein partners that might allow a more precise mRNA selection and binding.

## Discussion

Although multiple PABP homologues have been previously reported from different organisms, mainly metazoans and plants [25,27,29], a selective association of those with specific mRNAs targets has not been generally defined. This study identifies, for the first time in *Leishmania*, the association between one PABP homologue and a specific group of cytoplasmic mRNAs, those encoding ribosomal proteins. These constitute an important subset of mRNAs, encoding a large group of abundant proteins whose expression is in general tightly regulated [50]. Our data agrees with the known importance, seen in metazoans and other organisms, for the regulation of translation of ribosomal protein mRNAs. It also highlights the fact that the regulatory mechanisms associated with these mRNAs converge on interactions between their 5’ and 3’ ends. The description of how a specific PABP homologue can be involved in such mechanisms, through direct interactions with another RBP, further expands on the known roles seen for different PABPs in model organisms.

RNAi-mediated depletion of RBP23 has been previously shown to affect growth of the *T*. *brucei* bloodstream forms [51], with these cells displaying a gain-of-fitness phenotype during differentiation, but depletion does not seem to affect procyclic cells [52]. In *L*. *infantum*, studies with isobaric tagging methodology (iTRAQ) showed that RBP23 is significantly downregulated during differentiation and in the mature amastigote forms [53], suggesting that this protein is needed during exponential growth phases. Our data confirm the association of RBP23 with PABP1 and the EIF4E4/EIF4G3 complex, all three proteins known to enhance expression in tethering assays and also present in polysomes [33,42,43,54]. The mass-spectrometry results also indicate an association of RBP23 with ribosomes or polysomes in *Leishmania*, although in *T*. *brucei* its orthologue was not found associated with the polysomal fraction [54]. The RBP23 association with a translationally active complex based on EIF4E4/EIF4G3 is reinforced by the presence of EIF4AI in the immunoprecipitated fractions, with the lower EIF4AI enrichment possibly being either a consequence of greater abundance or a loose association with the complex.

Some of the additional proteins that co-precipitated with RBP23 highlight other likely functions, apart from translation. An early association with the mRNA is reinforced by the RBP23 co-precipitation with mRNA polyadenylation and nucleo-cytoplasmic transport factors. Both RBP23 and DRBD2, together with the two *T*. *brucei* PABP homologues, were also co-purified with TSR1, involved in splicing regulation, mRNA stability, and rRNA processing [55]. The CCR4-NOT deadenylase complex is an important mRNA regulator, interacting with transcription and translation activators and repressors and promoting mRNA decay [56]. The association with CCR4-NOT components may then indicate that RBP23 can remain bound to mRNA targets after their translation.

DRBD2 and two other proteins found here as co-precipitating partners, RBP12 and the uncharacterized LINF_200005800, all have *T*. *brucei* orthologues shown to reduce expression in tethering assays, while the orthologues of two other putative DRBD2 partners, PABP2 and PUF6, enhance expression [42,43]. *T*. *brucei* ZC3H31/34 and the LINF_210009700 orthologue (Tb927.10.2240) interact with the translational regulator MKT1 [44] while the ZC3H39/40 pair were identified as regulators of the mitochondrial respiratome [57]. The LINF_210009700 orthologue was further shown to interact with DRBD3, a cytoplasmic, stabilizing protein, which under oxidative stress remains bound to mRNA and concentrates in the nucleus, a typical behaviour of a protein involved in mRNA transport [58]. Our analysis also show a very relevant DRBD2 co-precipitation with proteins associated with the metabolism of mithochondrial RNAs. Although this is not supported by the current information regarding the DRBD2 localization [39], it also suggests a possible mitochondrial function. In all the evidence so far indicates that DRBD2 acts in different complexes with different contrasting functions.

In mammals, ribosomal proteins, as well as some translation factors, are encoded by mRNAs containing the 5’-terminal oligopyrimidine (TOP) sequence and which is recognized by the La-related protein 1 (LARP1) [50]. *Leishmania* RBP23, in contrast, is unlikely to specifically recognize its mRNA targets through 5’UTR TOP motifs, since all trypanosomatid mRNAs have the 5’ spliced leader sequence starting with two consecutive adenines [59]. Other sequence motifs within the 5’UTRs of mRNAs encoding ribosomal proteins and which could mediate specific recognition are unlikely, due to their small sizes [49]. These small 5’UTRs, however, raise another issue regarding the need for an eIF4F complex, with an associated eIF4A helicase. Once thought to be mainly required for the translation of mRNAs having structured 5’UTRs, eIF4A has been recently shown to be required for the translation of most mRNAs, in both yeast and mammals [60,61]. The protozoan *Giardia lamblia*, which lacks the eIF4G subunit of eIF4F [62], is also characterized by mRNAs having very short 5’UTRs [63] which nevertheless seem to require eIF4A, plus one of two *Giardia* eIF4Es, to mediate their interaction with the translation pre-initiation complex [64]. These reports are consistent with a strict requirement for eIF4A, and the trypanosomatid EIF4AI, for the translation of mRNAs regardless of the size of their 5’UTRs.

Generally, mRNAs encoding ribosomal proteins have their translation differentially regulated through diverse mechanisms. In yeasts, the phosphorylation of an activator (Ifh1) and a repressor (Crf1) controls the transcription of genes encoding these mRNAs [65]. RNA sequencing analysis demonstrated that they are also especially enriched with the closed loop translation initiation components, eIF4E and both isoforms of eIF4G [66]. In mammals, the LARP1 protein, when associated with the TOP motif and the 5’ *cap*, acts as a translational repressor that binds to PABP and consequently form a translation-inactive mRNA loop. The mammalian target of rapamycin complex 1 (mTORC1), a kinase complex, phosphorylates LARP1 and releases it from 5’TOP mRNAs. This leads to the eIF4F assembly, since mTORC1 also controls phosphorylation of 4E-BP1, thereby allowing eIF4G to bind to eIF4E and recruit the 43S complex to start translation [50,67,68].

**Fig 8:**
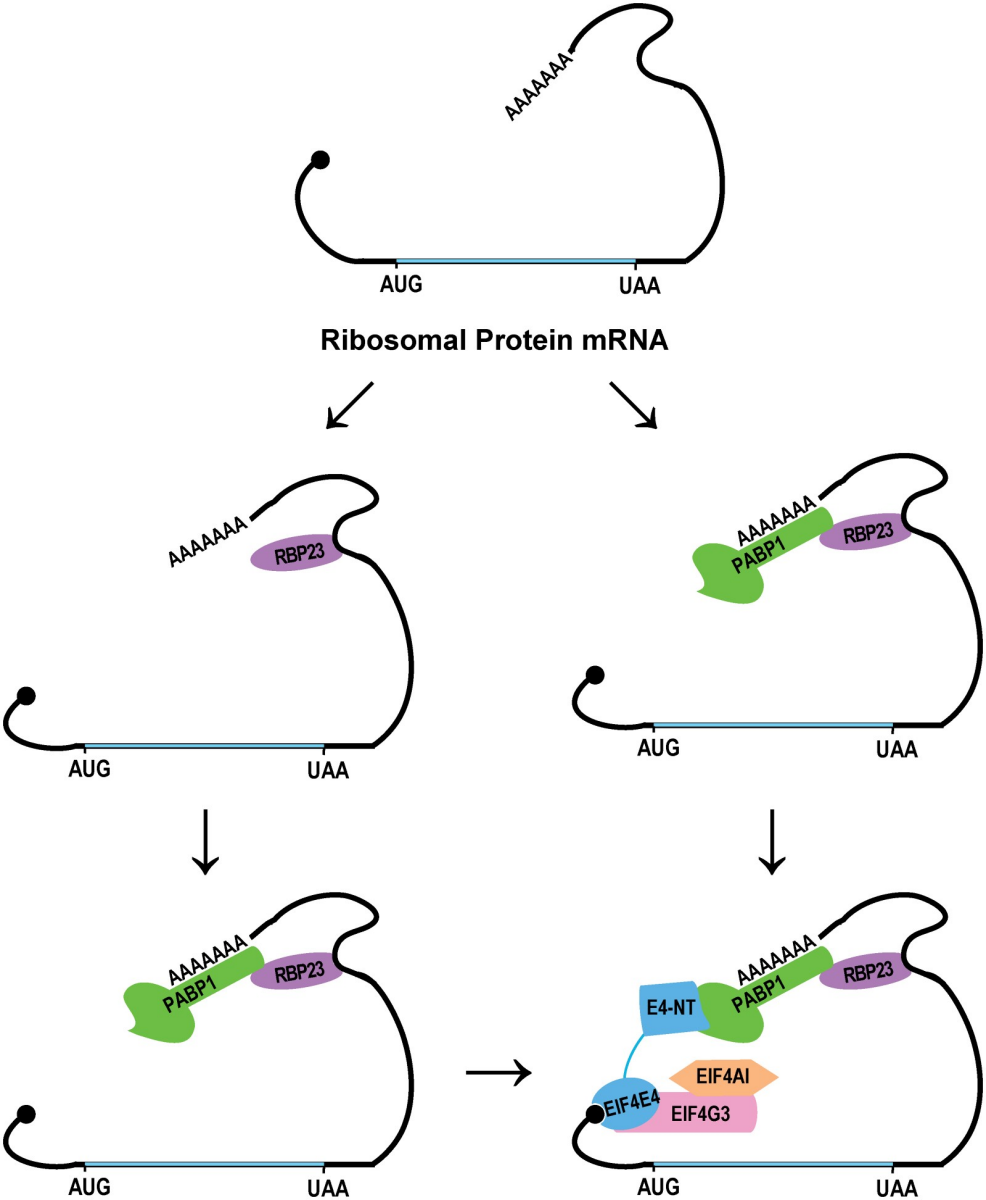
Proposed role for RBP23 in mediating the association of mRNAs encoding ribosomal proteins with PABP1 and the EIF4E4/EIF4G3/EIF4AI complex. According to the proposed model, RBP23 recognizes target mRNAs through motifs within their 3’UTRs. It might recognize these mRNAs on its own or already bound to PABP1. PABP1 then mediates the recruitment of the translation initiation complex EIF4E4/EIF4G3/EIF4AI. It is also possible that PABP1 might already be bound to the complex prior to mRNA recognition but this is not shown. Both PABP1 and EIF4E4 are also phosphorylated during exponential growth, allowing for a more refined regulation of their activity and, presumably, the translation of the ribosomal protein mRNAs.

Both *Leishmania* PABP1 and EIF4E4 are simultaneously phosphorylated during exponential growth [33,36], possibly associated with activation of translation of their target mRNAs. RBP23 may help recruit PABP1 and the EIF4E4/EIF4G3 based complex to selected mRNAs, mainly mRNAs encoding ribosomal proteins, to promote their translation. Here, we propose a model where the trypanosomatid RBP23 binds to common 3’UTRs motifs shared by these mRNAs and mediates their association with PABP1 (Fig 8). The RBP23 specificity for the ribosomal protein mRNAs, in comparison to a broader range of mRNAs associated with PABP1, and the specificity of the later to poly(A) sequences, strongly implicates RBP23 as the protein responsible for mRNA recognition, on its own or dependent on additional protein partners. Thus, RBP23 may mark the ribosomal protein mRNAs as targets for translation mediated by the complex PABP1/EIF4E4/EIF4G3/EIF4AI. Preferential binding of this eIF4F complex to the mRNAs encoding ribosomal proteins would direct them to a translation pathway distinct from most other cellular mRNAs, perhaps avoiding competition and in agreement with these mRNAs behaving distinctively than most other messages during translation [69]. It would also allow their translation to be specifically regulated, presumably involving the phosphorylation of EIF4E4 and PABP1, an event already shown to be cell-cycle regulated [33,36,38]. Thus, according to the proposed model, translation regulation of the mRNAs encoding ribosomal proteins in trypanosomatids would also require interactions involving proteins bound to both the 5’ (EIF4E4) and 3’ (PABP1) ends of the mRNAs, as seen for the translation regulation of these mRNAs in mammals. It remains to be seen whether the EIF4E4/PABP1 interaction may also lead to a translation-inactive loop that may be enhanced or abolished by their simultaneous phosphorylation.

## Materials and methods

### Plasmid constructs and DNA manipulations

*L*. *infantum* genomic DNA from the MHOM/MA/67/ITMAP-263 strain was isolated using DNAzol (Life Technologies) following the manufacturer’s instructions. Full length *RBP23*, *DRBD2*, *PABP2* and *PABP3* genes were amplified using primers flanked by BamHI and HindIII restriction sites (listed in S8 Table) and cloned into the BamHI-HindIII sites of a modified version of the *Leishmania* expression vector pSPBT1YNEOα [70]. The modification consisted of the insertion of a 27 nucleotide element encoding the HA epitope (YPYDVPDYA) immediately after the coding sequence and prior to the translation stop codon. The *RPB23* and *DRBD2* genes were also subcloned into the BamHI-HindIII sites of the pET-21a vector (Novagen), while the *PABP2* and *PABP3* genes were further subcloned into the same sites of a modified pGEX4T3 expression vector (GE Healthcare) having an added HindIII site.

### Parasite growth, transfections and expression analysis

*Leishmania major* MHOM/IL/81/Friedlin and *Leishmania infantum* MHOM/MA/67/ITMAP-263 promastigotes were cultured in Schneider’s insect medium supplemented with 1% Penicillin-Streptomycin, 10% heat-inactivated Fetal Bovine Serum and 2% hemin at pH 7.2, 25°C. Transfection of the circular plasmids was carried out by electroporation, with exponentially grown cells resuspended in HEPES-NaCl buffer (21 mM HEPES pH 7.05, 137 mM sodium chloride, 5 mM potassium chloride, 0.7 mM disodium phosphate, 6 mM glucose), incubated with the plasmid DNA and submitted to a pulse of 450 V, 500 μF on the Gene Pulser Xcell electroporation system (Bio-Rad). Transfected cells were selected with G418 (20 μg/ml, Sigma). For the expression analysis of cells expressing the HA-tagged proteins, late exponentially grown *L*. *infantum* cultures were harvested and resuspended directly into denaturing SDS-PAGE sample buffer, submitted to 15% SDS-PAGE and blotted with an anti-HA mouse monoclonal antibody (100 ng/ml, Applied Biological Materials).

### Sequence analyses

Secondary structure and domain predictions for RBP23 were performed using the Phyre2 automatic fold recognition server [71]. Domains were also identified using the InterPro tool available at http://www.ebi.ac.uk/interpro/search/sequence/. The 3’ UTR analyses were performed through MEME [72] using 300 nucleotides immediately after the stop codon from the top 20 mRNA sequences bound to the *L*. *infantum* RBP23-HA and DRBD2-HA [48]. The already defined 3’ UTRs of *L*. *donovani* orthologues were also retrieved and analysed. Default parameters were used to detect the motifs, using any number of sites per sequence and a width ranging between six and 50 nucleotides.

### Cytoplasmic extract preparation

For the RNA sequencing analysis, whole *Leishmania major* (MHOM/IL/81/Friedlin) cytoplasmic extracts were produced from late exponentially grown promastigote cultures harvested and washed once in ice cold PBS, prior to resuspension in IPM1 buffer [100 mM KCl, 5 mM MgCl_2_, 10 mM HEPES, protease inhibitors (Roche), RNAseOUT and 0.5% IGEPAL CA-630 (Sigma)] to a concentration of 1 to 2x10^9^ cells/ml. The resuspended cells were left for 10 minutes on ice and then centrifuged for 10 more minutes at 17.000 g, 4°C, with the sediment discarded and the supernatants, the cytoplasmic extracts, aliquoted and stored at -80°C. Whole cytoplasmic extracts from *L*. *infantum* wild-type and strains expressing various HA-tagged proteins were generated after cell lysis using nitrogen cavitation [73]. Briefly, late exponentially grown *L*. *infantum* promastigotes were harvested and washed once in ice cold PBS, followed by resuspension in HEPES-lysis buffer (20 mM HEPES-KOH pH7.4, 75 mM potassium acetate, 4 mM magnesium acetate, 2 mM DTT, supplemented with EDTA-free protease inhibitors from Roche) to a concentration of 1 to 2x10^9^ cells/ml. The resuspended cells were transferred into the cavitation chamber of the cell disruption vessel (Parr Instruments) and incubated at 4°C under 70 bar pressure for 40 minutes, followed by rapid decompression/lysis. The lysates were submitted to centrifugation, as described above, with the supernatants aliquoted and stored likewise.

For mass-spectrometry analysis, total cytoplasmic extracts from *L*. *infantum* expressing HA-tagged PABP1, PABP2, PABP3, as well as a phosphorylation PABP1 mutant (TP-SP), were generated after lysis using acid-washed glass beads, 425–600 μm *(Sigma)*, as described previously [33]. For parasites expressing HA-tagged RBP23 and DRBD2, and wild-type controls, total cytoplasmic extracts were obtained after cell lysis through nitrogen cavitation, as described above. None of the cytoplasmic extracts used for the immunoprecipitation assays and subsequent mass spectrometry analysis were treated with RNase.

### Immunoprecipitation studies

For immunoprecipitations (IPs) with the HA-tagged proteins, whole *L*. *infantum* cytoplasmic extracts from wild type or recombinant HA-tagged strains were mixed with Pierce Anti-HA magnetic beads as per manufacturer’s protocol. Briefly, 0.2 mg of the beads were washed three times with PBS followed by the incubation with 0.5 ml of cytoplasmic extracts (equivalent to ~5x10^8^ to 10^9^ cells) for 1 h at 4°C. After removal of the depleted supernatant, the beads were washed three times with PBS and the bound antigen-antibody complexes eluted in SDS-PAGE sample buffer. Roughly 20% of the IPs were then analysed through SDS-PAGE and western-blotting using antibodies against the HA-tag to confirm the efficiency of the precipitation reaction. Immunoprecipitations were performed in triplicates and duplicates for RNA-sequencing and mass spectrometry, respectively.

For IP assays against native PABPs, exclusively for RNA-sequencing, we used approximately 0.1 mg of protein A sepharose beads, whole *L*. *major* cytoplasmic extracts and previously described affinity purified antibodies raised against each of the three *Leishmania* PABPs [30]. After a first wash with PBS, the beads were incubated with the antibodies (50 μL) in PBS overnight at 4°C, washed again with PBS and then incubated with the whole *L*. *major* extracts (equivalent to 1x10^9^ cells) for 1 h, at 4°C, in the presence of RNAseOUT. After spinning for 2 minutes at 600 g, 4°C, the supernatant was removed and the beads washed three times sequentially with IPM1 buffer containing 1% IGEPAL CA-630 and RNAseOUT.

### RNA extraction and cDNA library construction

For the RNA sequencing of ligands bound to the HA-tagged proteins, RNA was extracted with the RNAeasy Mini Kit (QIAGEN) from three independent immunoprecipitation experiments. These were carried out with different batches of cytoplasmic extracts derived from wild-type *L*. *infantum* and transfected cells expressing HA-RBP23, HA-DRBD2 and the three HA-tagged PABPs. The RNA samples were quantified by Qubit RNA HS Assay Kit (Thermo Fisher) using to read the concentration the Qubit 2.0 Fluorometer. Between 0.1 to 4 μg of total RNA was used to construct the cDNA libraries with the TrueSeq Stranded mRNA Library Prep Kit (Illumina). The libraries were validated quantitatively through qPCR using the KAPA Library Quantification Kit and qualitatively by visualization in agarose gel. Finally, the libraries were normalized and pooled prior to sequencing using the MiSeq Reagent Kit v3, 150 cycle (Illumina). Similar RNA extractions were performed for the *L*. *major* IPs carried out with the native anti-PABP affinity purified antibodies, and total RNA was used to prepare cDNA libraries using the SOLiD Whole Transcriptome Analysis Kit followed by evaluation with an Agilent Bioanalyzer (Agilent). The cDNA libraries were used for clonal amplification according to the SOLiD Full-Scale Template Bead preparation protocol and sequenced with the SOLiD4 System (Applied Biosystems).

### RNA sequencing analysis and data normalization

RNA-seq reads obtained from SOLiD data were mapped against the *Leishmania major* Friedlin genome assembly version 8.1 available at the TriTrypDB database by the SHRiMP software version 2.2.3 [74], with default parameters. All mappings whose score was higher than 350 were considered for further analyses, where all samples were normalized and the differential expression was assessed by the EdgeR package [75], included in the Bioconductor package version 2.6 [76]. mRNAs co-precipitated with each HA-tagged protein were analysed with the following bioinformatic tools: (1) FastQC, to evaluate the quality of the sequences (https://www.bioinformatics.babraham.ac.uk/projects/fastqc/); (2) Trimmomatic [77], version 0.36, to remove the adapters and the low-quality sequences; (3) STAR [78], to map the reads on the *L*. *infantum* genome and count reads associated with each gene; (4) DEseq2 Galaxy version [79], to statistically compare the samples. Genes were considered enriched when a four-fold increase (log_2_ ratio > = 2) was observed when compared to the negative control, with FDR of 0.01 or 0.05 considered, respectively, for the IPs with the native proteins or HA-tagged ones. mRNAs enriched with the different proteins were then classified according to a modified list of gene ontology (GO) functional terms. For the RBP23 mRNA analysis, the transcript sequence containing the 3’ UTR and the transcript sequence with HA, but without the 3’ UTR, was used as reference. Bowtie2, a tool for aligning sequencing read [80] was used to generate the.bam files, which were viewed in IGV, integrative genomics viewer, a visualization tool [81,82].

### Mass-spectrometry analysis

For protein digestion and mass-spectrometry, IPs of HA-tagged PABP1, TP-SP, PABP2 and PABP3 were analysed by the Proteomics platform of the Quebec Genomics Center at the CHU de Quebec Research Center-Université Laval (http://www.crchudequebec.ulaval.ca/en/services/proteomics/about-us/), as described [83]. The results of two independent experiments were analysed by the Scaffold Proteome software used to validate the protein identification based on the *L*. *infantum* genome. Only proteins identified with >2 peptide and a probability of >80.0% were considered. Eluted proteins from IP of HA-tagged RBP23 and DRBD2, also from two independent experiments, each with two sets of negative controls, were submitted to the mass spectrometry facility P02-004 at the Carlos Chagas Institute–Fiocruz-PR. The samples were loaded into 15% SDS-PAGE gels and allowed to migrate into the resolving gel, when the electrophoresis was interrupted prior to protein fractionation. Gel slices containing the whole IP products were then excised and submitted to an in-gel tryptic digestion and mass spectrometry analysis and validation was performed as previously described [47]. Spectra were searched against the *L*. *infantum* protein sequence database (*L*. *infantum* JPCM5, version from March 29, 2016, available at TriTrypDB; https://tritrypdb.org/). To normalize the data from the IPs with the HA-tagged RBP23 and DRBD2, a first normalization was performed based on the sum of the intensities for each replicate, using the highest sum to normalize the remaining samples. For each co-precipitated polypeptide, for the negative control and both RBPs, the averages derived from the normalized intensities were calculated. These averages were then used to calculate the ratio between the values from the IPs with each HA-tagged protein and those control IPs using extracts from non-transfected cells (enrichment ratio).

### *In vitro* pull-down assays

Pull-down assays were performed using Glutathione Sepharose 4B beads (GE Healthcare) as well as GST-tagged and ^35^S-labeled recombinant proteins, as described previously [36,84]. GST-tagged PABPs and the GST control, whose genes were cloned into the pGEX4T3 expression vector, were expressed in *Escherichia coli*, affinity purified, immobilized on the beads and incubated with ^35^S-labeled RBP23 and DRBD2. The labeled proteins were produced after linearization with HindIII of the corresponding constructs in the pET21a plasmid, followed by transcription with T7 RNA polymerase in the presence of the cap analogue and translation in the rabbit reticulocyte lysate (Promega or Ambion) supplemented with ^35^S-methionine (Perkin Elmer). The labeled signals were visualized by autoradiographic films exposed unto 15% SDS-PAGE gels.

## Supporting information

S1 Fig Analysis of mRNA populations associated with the three native PABPs in *Leishmania major*. Upregulated transcripts in at least two of three available RNA-seq datasets (SOLiD sequencing) were manually classified and grouped using the gene ontology (GO) terms according to their molecular function. **A)** mRNA groups associated with PABP1, PABP2 and PABP3 from *L*. *major*. All mRNAs enriched at least 2-fold more than the negative control are represented; **B)** Bar chart representing the enrichment values only of mRNAs co-immunoprecipitated with the three *L*. *major* PABPs that were enriched at least 4-fold. The mRNAs with the same names indicate different transcripts encoding proteins with the same name but whose genes localize to different chromosomes.
(TIF)

S2 Fig Expression analysis of the HA-tagged *L*. *infantum* PABPs. Western-blot analysis of exponentially grown *L*. *infantum* cell lines expressing the HA-tagged *L*. *infantum* PABPs. Approximate molecular weights of 63, 65 and 61 kDa were seen for PABP1-HA, PABP2-HA and PABP3-HA, respectively.
(TIF)

S3 Fig Volcano Plot comparing mRNA content of *L*. *infantum* PABPs RNA-seq datasets. Axis Y represents the statistical test significance 0.05 (>0.05 red and <0.05 black) and the axis X represents the genes that are at least 4-fold up- or down-regulated (log_2_ fold change >2); Δ means a very high value to be ranked. (**A**) PABP2xPABP1; (**B**) PABP3xPABP1; and (**C**) PABP3xPABP2.
(TIF)

S4 Fig Comparison of RNA-seq datasets from RNA-immunoprecipitation studies using the three *Leishmania infantum* PABPs. The mRNAs associated to each PABP homologue are indicated in a Venn diagram from their respective RNAseq datasets.
(TIF)

S5 Fig Summary of RBPs co-purifying with the *L*. *infantum* HA-tagged PABPs. RBPs shown here are differentially co-precipitated with PABP1 and PABP2/3 in two independent immunoprecipitation (IP) assays. The values represent the number of peptide hits from proteins found with each PABP. Only proteins identified with two or more peptides in a minimum of two replicates and with a probability >80.0% were considered. TP-SP represents a PABP1 phosphorylation mutant (described in [33]), used as a second PABP1 sample, since it was presumed that the mutations would not impact on the interactions with major binding partners.
(TIF)

S6 Fig Localization of the most-relevant motifs found on the topmost 10 mRNAs enriched with the *L*. *infantum* HA-tagged RBP23 or DRBD2. **A)** U-rich motifs found within the 3’ UTRs of the RBP23 associated mRNAs; **B)** Motifs having UG-repeats found within the 3’ UTRs of the DRBD2 associated mRNAs.
(TIF)

S1 Table mRNAs specifically co-precipitated with the three *L*. *major* PABP homologues. (XLSX)

S2 Table mRNAs specifically co-precipitated with the three, HA-tagged, *L*. *infantum* PABP homologues. (XLSX)

S3 Table Full set of protein partners specifically co-precipitated with the HA-tagged RBP23. (XLSX)

S4 Table Top-most protein partners specifically co-precipitated with the HA-tagged RBP23. (XLSX)

S5 Table Full set of protein partners specifically co-precipitated with the HA-tagged DRBD2. (XLSX)

S6 Table Top-most protein partners specifically co-precipitated with the HA-tagged DRBD2. (XLSX)

S7 Table mRNAs specifically co-precipitated with the HA-tagged *L*. *infantum* RBP23 and DRBD2. (XLSX)

S8 Table Oligonucleotides used as primers for the amplification and cloning of the PABP2, PABP3, RBP23 and DRBD2 genes. (XLSX)
