# Supplementary figures and images for: Identification of novel proteins and mRNAs differentially bound to the Leishmania Poly(A) Binding Proteins reveals a direct association between PABP1, the RNA-binding protein RBP23 and mRNAs encoding ribosomal proteins
Source: PLoS Negl Trop Dis. 2021 Oct 27;15(10):e0009899. doi: 10.1371/journal.pntd.0009899 (PMC8575317; doi:10.1371/journal.pntd.0009899)

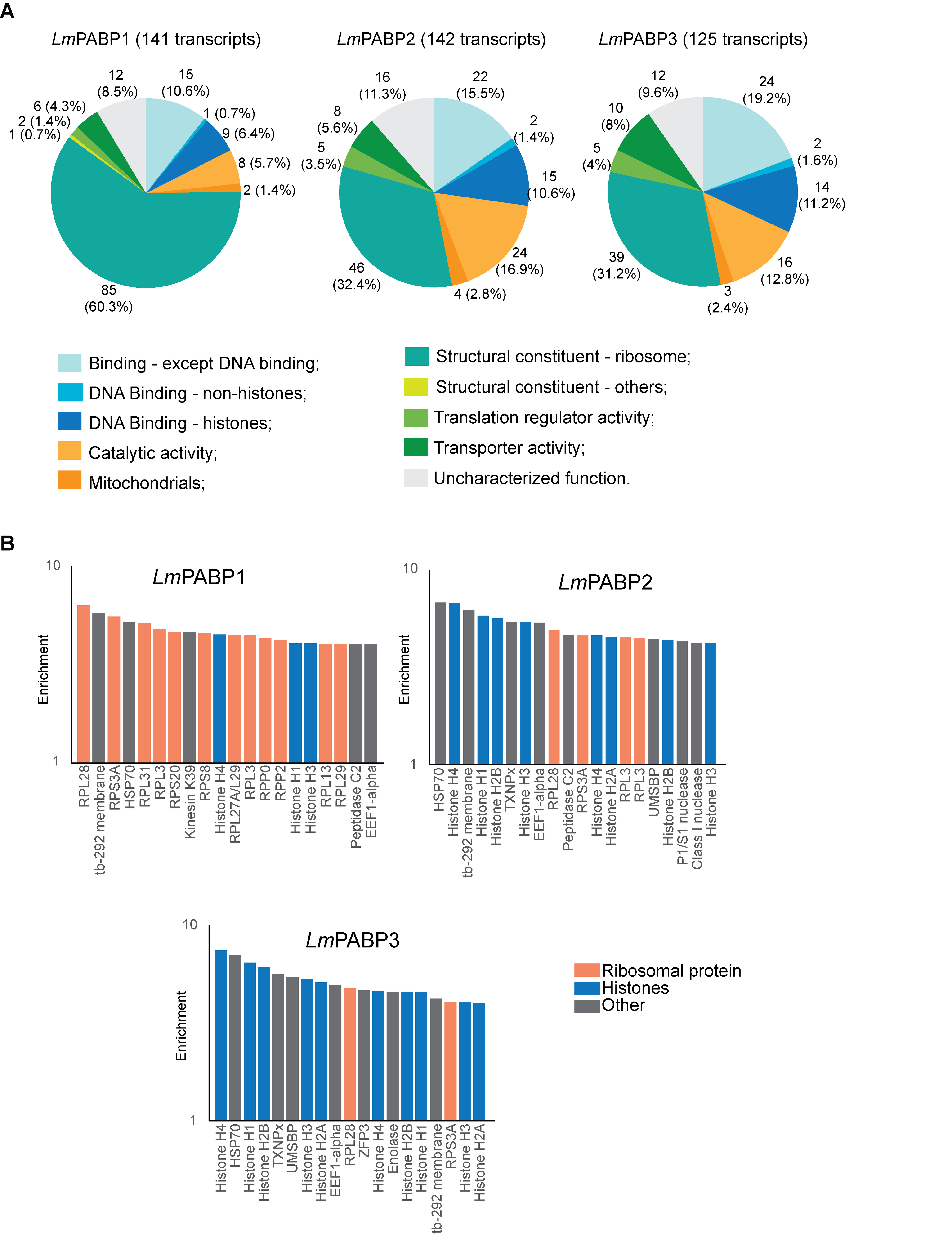

Supplement: S1 Fig — Upregulated transcripts in at least two of three available RNA-seq datasets (SOLiD sequencing) were manually classified and grouped using the gene ontology (GO) terms according to their molecular function. A) mRNA groups associated with PABP1, PABP2 and PABP3 from L. major. All mRNAs enriched at least 2-fold more than the negative control are represented; B) Bar chart representing the enrichment values only of mRNAs co-immunoprecipitated with the three L. major PABPs that were enriched at least 4-fold. The mRNAs with the same names indicate different transcripts encoding proteins with the same name but whose genes localize to different chromosomes. (TIF) [file pntd.0009899.s001.tif]

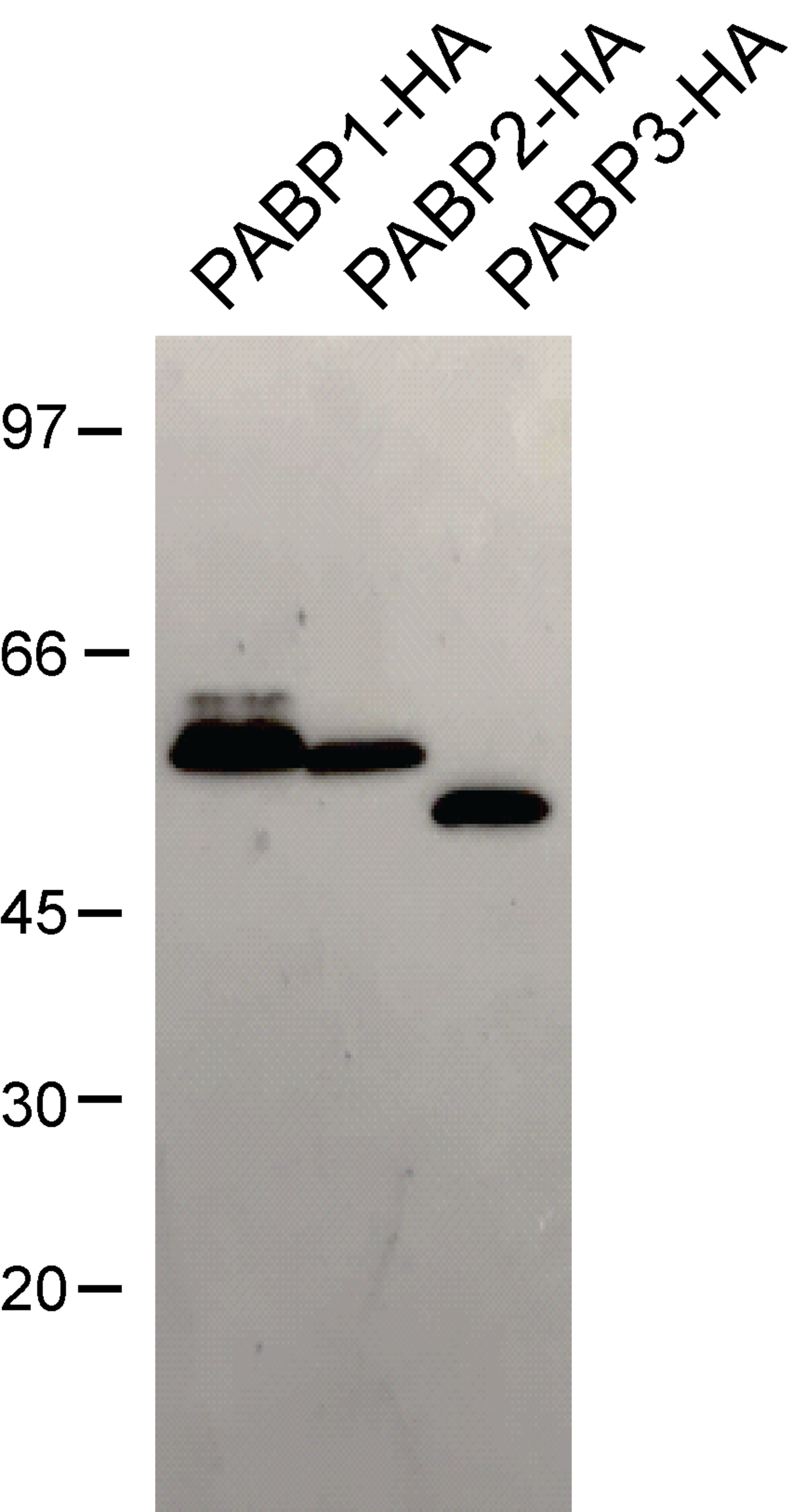

Supplement: S2 Fig — Western-blot analysis of exponentially grown L. infantum cell lines expressing the HA-tagged L. infantum PABPs. Approximate molecular weights of 63, 65 and 61 kDa were seen for PABP1-HA, PABP2-HA and PABP3-HA, respectively. (TIF) [file pntd.0009899.s002.tif]

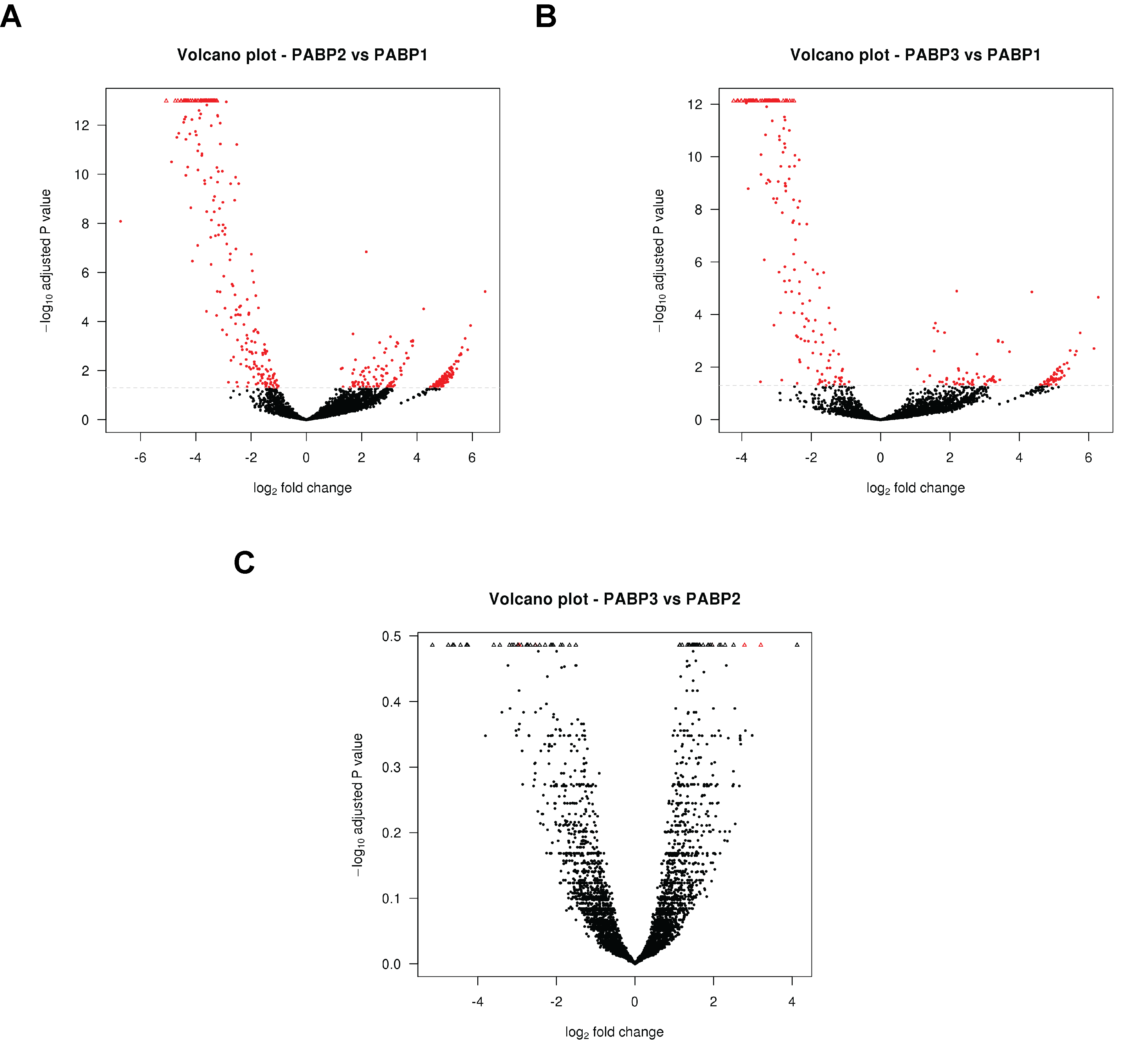

Supplement: S3 Fig — Axis Y represents the statistical test significance 0.05 (>0.05 red and <0.05 black) and the axis X represents the genes that are at least 4-fold up- or down-regulated (log2 fold change >2); Δ means a very high value to be ranked. (A) PABP2xPABP1; (B) PABP3xPABP1; and (C) PABP3xPABP2. (TIF) [file pntd.0009899.s003.tif]

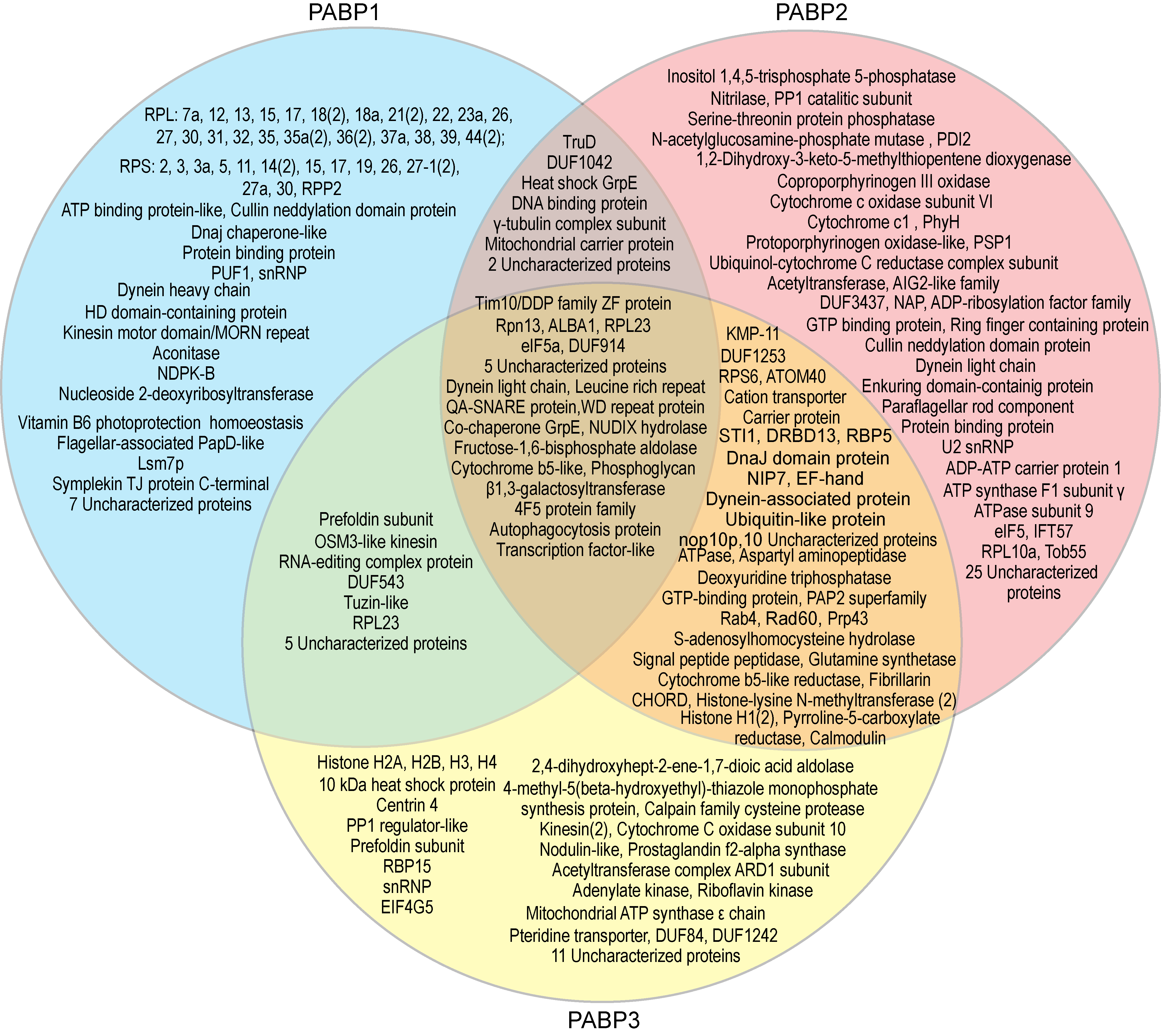

Supplement: S4 Fig — The mRNAs associated to each PABP homologue are indicated in a Venn diagram from their respective RNAseq datasets. (TIF) [file pntd.0009899.s004.tif]

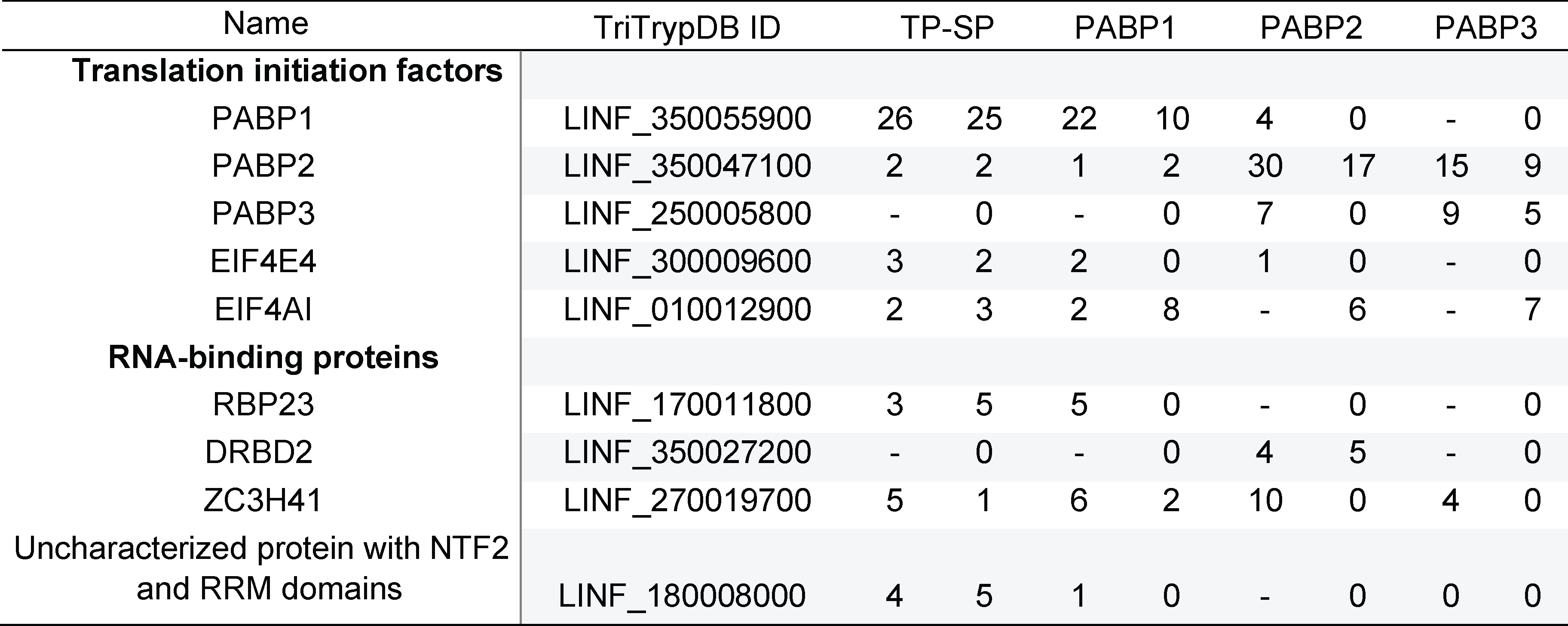

Supplement: S5 Fig — RBPs shown here are differentially co-precipitated with PABP1 and PABP2/3 in two independent immunoprecipitation (IP) assays. The values represent the number of peptide hits from proteins found with each PABP. Only proteins identified with two or more peptides in a minimum of two replicates and with a probability >80.0% were considered. TP-SP represents a PABP1 phosphorylation mutant (described in [33]), used as a second PABP1 sample, since it was presumed that the mutations would not impact on the interactions with major binding partners. (TIF) [file pntd.0009899.s005.tif]

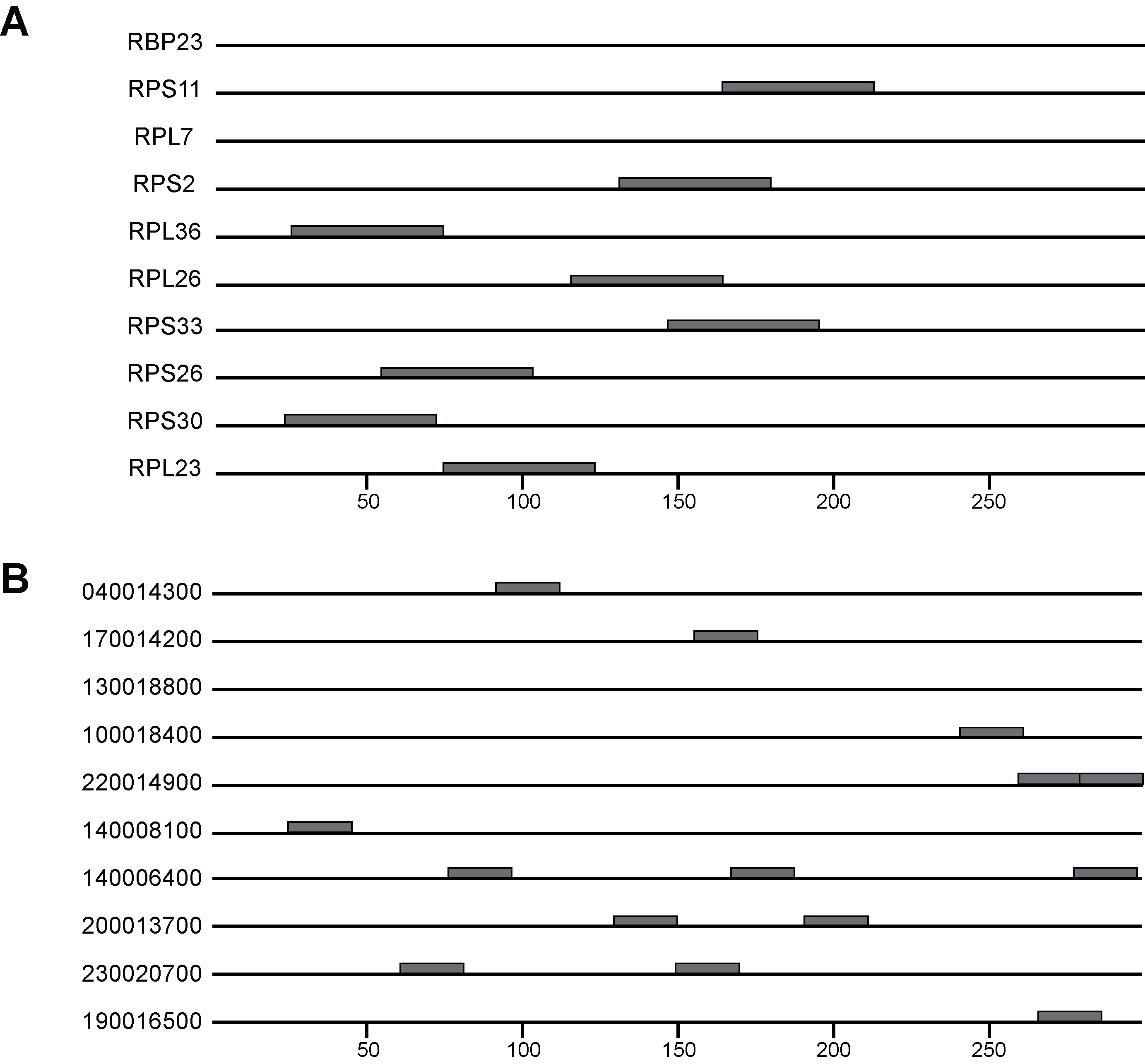

Supplement: S6 Fig — A) U-rich motifs found within the 3’ UTRs of the RBP23 associated mRNAs; B) Motifs having UG-repeats found within the 3’ UTRs of the DRBD2 associated mRNAs. (TIF) [file pntd.0009899.s006.tif]
